# Fracture profiles of a 4-year cohort of 266,324 first incident upper extremity fractures from population health data in Ontario

**DOI:** 10.1186/s12891-021-04849-7

**Published:** 2021-11-29

**Authors:** Joy C. MacDermid, J. Andrew McClure, Lucie Richard, Kenneth J. Faber, Susan Jaglal

**Affiliations:** 1 School of Physical Therapy, Western University, London, ON Canada; 2 ICES Western, London, ON Canada; 3 Department of Surgery, Western University, London, ON Canada; 4 Hand and Upper Limb Centre, St. Joseph’s Health Centre, 268 Grosvenor St, London, ON Canada; 5 Department of Physical Therapy, University of Toronto, Toronto, ON M5G 1V7 Canada

**Keywords:** Fractures, Epidemiology, Upper extremity, Distal radius, Proximal humerus, Social deprivation, Comorbidity, Nerve injury, Tendon injury, Sex differences, Scaphoid

## Abstract

**Background:**

Understanding the profiles of different upper extremity fractures, particularly those presenting as a 1st incident can inform prevention and management strategies. The purpose of this population-level study was to describe first incident fractures of the upper extremity in terms of fracture characteristics and demographics.

**Methods:**

Cases with a first adult upper extremity (UE) fracture from the years 2013 to 2017 were extracted from administrative data in Ontario. Fracture locations (ICD-10 codes) and associated characteristics (open/closed, associated hospitalization within 1-day, associated nerve, or tendon injury) were described by fracture type, age category and sex. Standardized mean differences of at least 10% (clinical significance) and statistical significance (*p* < 0.01) in ANOVA were used to identify group differences (age/sex).

**Results:**

We identified 266,324 first incident UE fractures occurring over 4 years. The most commonly affected regions were the hand (93 K), wrist/forearm(80 K), shoulder (48 K) or elbow (35 K). The highest number of specific fractures were: distal radius (DRF, 47.4 K), metacarpal (30.4 K), phalangeal (29.9 K), distal phalangeal (24.4 K), proximal humerus (PHF, 21.7 K), clavicle (15.1 K), radial head (13.9 K), and scaphoid fractures (13.2 K). The most prevalent multiple fractures included: multiple radius and ulna fractures (11.8 K), fractures occurring in multiple regions of the upper extremity (8.7 K), or multiple regions in the forearm (8.4 K). Tendon (0.6% overall; 8.2% in multiple finger fractures) or nerve injuries were rarely reported (0.3% overall, 1.5% in distal humerus). Fractures were reported as being open in 4.7% of cases, most commonly for distal phalanx (23%). A similar proportion of females (51.5%) and males were present in this fracture cohort, but there were highly variant age-sex profiles across fracture subtypes. Fractures most common in 18–40-year-old males included metacarpal and finger fractures. Fractures common in older females were: DRF, PHF and radial head, which exhibited a dramatic increase in the over-50 age group.

**Conclusions:**

UE fracture profiles vary widely by fracture type. Fracture specific prevention and management should consider fracture profiles that are highly variable according to age and sex.

**Supplementary Information:**

The online version contains supplementary material available at 10.1186/s12891-021-04849-7.

## Introduction

Clinicians and health systems must manage the large volume of upper extremity fractures that present for care. Awareness of classic fracture presentations informs health promotion and clinical management strategies. Some aspects of fracture epidemiology have been well investigated, while in other areas gaps remain. Upper extremity fractures like distal radius fractures (DRF) and proximal humerus fractures (PHF) that occur from a fall from level ground are classified as fragility fractures and considered an early indicator of compromised bone health [1]. Other fractures are more related to greater amounts of trauma. Previous studies that have focused on the epidemiology of upper extremity fractures have focused on more common fractures [2], particularly the wrist [3], hand [4] and proximal humerus fractures [5, 6]. Distal radius fractures (DRF) occur in about 6% of males and 33% of females [2, 3] at some point in their lifetime. Some common upper extremity fractures have epidemiologic data reported, while other types of upper extremity fractures have been rarely described. Further, most fracture literature has focused on all fractures, rather than first incident fractures. For certain fractures, particularly those related to osteoporosis, the risk of recurrent fractures is much higher than an initial fracture and is likely to have a different risk profile. A systematic review concluded that a prior wrist fracture was associated with approximately a doubling of risk for subsequent fractures [4]. Hand fracture rates have been described in terms of rates, costs and occupational impacts [5, 6]. Proximal humeral fractures (PHF) [1, 4], have been linked to compromised bone health [7], fragility and frailty [8].

High trauma fractures, compromised bone (bone diseases including osteopenia or osteoporosis) and frailty fractures are different from a prevention and management perspective. Frailty is an independent predictor of fracture, disability, and falls in females aged 55 and older in 10 countries [9] which is evident in sex differences in fracture incidence in these age groups. Although few studies have directly compared the profiles of different upper extremity fractures the literature to date indicates that different profiles should be expected based on age and sex [10].

Previous studies of upper extremity fractures have focused on only a few fracture types [11], sampled in only one city [12], only included patients over 60 or 65 [13, 14], excluded trauma [14] or included both first and recurrent fractures. Since a 1st incident fracture is the one where differentiating trauma, bone compromise and frailty fractures from each other is least clear, and predicting future trajectories is least certain; this complicates prevention and case management. Once a pattern of recurrent fractures is evident, compromised bone health is more recognizable. Description of the profiles of a 1st incident fracture is a 1st step to informing future research on early prevention strategies and can assist with identifying how typical an individual presenting with a fracture is, as part of the overall clinical decision-making. Therefore, the purposes of this study were to describe a cohort of adult patients having their first upper extremity fracture in terms of injury patterns: fracture numbers, fracture types (location, % open), associated nerve or tendon injuries, injury seasonal patterns and hospitalization rates.

## Methods

### Design

Retrospective cohort using administrative data.

#### Data sources and extraction

We conducted this study using health administrative data in Ontario, Canada’s largest province. Ontario administers health care using a single-payer, universally accessible system. As such, administrative datasets provide a population-level view of healthcare in the province. Datasets were linked using unique encoded identifiers and analyzed at the Institute for Clinical Evaluative Sciences (ICES). Details of ICES datasets are in Appendix A. Under Ontario law (Personal Health Information Protection Act, Ontario Regulation 329/04), ICES is named as a prescribed entity and can receive and use health information without consent for the purposes of compiling and analyzing statistical information about the health care system in Ontario. ICES goes to great lengths to protect privacy and is recognized as an international leader in maintaining the security of health information.

Healthcare encounters were recorded in multiple record-level, administrative datasets in the ICES Data Repository, and encrypted patient-specific identifiers (ICES-specific key number [IKN]) were used to link the administrative datasets. Patient data records were obtained from Canadian Institute for Health Information’s (CIHI) Discharge Abstract Database and Same Day Surgery for inpatient hospital care, National Ambulatory Care Reporting System for emergency visits, Ontario Health Insurance Program for physician billing data, and other data containing sociodemographic information (ICES Registered Persons Database and Statistics Canada Census). Additionally, ICES-developed disease cohorts were used to identify rheumatoid arthritis (Ontario Rheumatoid Arthritis Database) [15, 16] and diabetes (Ontario Diabetes Database). Fracture codes based on ICD-10 are listed in Appendix B.

#### Fracture identification/description

We included all patients over 18 years of age who visited the emergency room with an upper extremity fracture from January 1, 2013 to December 31. 2017. Only the first such event was eligible for inclusion. To ensure all fracture-related diagnoses were captured, we appended diagnoses reported during ER visits and hospital admissions within 5 days of the initial ER visit. We excluded non-Ontario residents and those with evidence of an upper extremity fracture, as an adult, within the previous 10-years (previous pediatric fractures were permitted). To help make sure we identified all previous fractures, we also excluded individuals who were not eligible for healthcare services in Ontario for this full 10-year look-back period.

#### Fracture profiles

Fractures were described by region, specific types, open/closed, season and whether a tendon or nerve injury was identified. Fracture ICD-10 codes were grouped together based on clinical relevance; compiling open and closed fractures, and codes where multiple codes were used for the same type: clavicle, scapula, proximal humerus, humerus shaft, distal humerus, head/neck of the radius, distal radius fractures, carpal bones other than the scaphoid, or first metacarpal (ICD-10 codes multiple areas on the first metacarpal but does not differentiate other metacarpals), proximal/other phalangeal fractures. Other specific fractures were identified by single ICD-10 codes including olecranon, coronoid, Monteggia, ulnar shaft, scaphoid, and distal phalanx fracture (but combined across open and closed options). ICD-10 codes describing multiple concurrent fractures of the shoulder, radius and ulna, forearm, metacarpals, or digits were combined and identified as multiple fractures within those areas. Where concurrent fractures codes were from different regions (e.g. shoulder and hand), this was coded as fracture in more than 1 region. (Appendix B).

Season was divided into four 3-month intervals, starting in January, to make the exposure time relatively similar across time intervals. Rurality was defined as residing in a community of less than 10,000 people based on the address designation classification by Statistics Canada.

Nerve injury was grouped as a major nerve injury when codes for specific major nerves in the upper arm (ulnar, median, radial, axillary, musculocutaneous), forearm (median, ulnar, radial), or wrist (median/ulnar) were identified; and as any nerve injury by collapsing all specific and non-specific nerve codes for each of these 3 areas.

#### Patient profiles

We constructed demographic and fracture characteristic profiles of the cohort overall and of major fracture subtypes. Age was classified based on clinically relevant subgroups: ages 18 to 40 (young adults), 41 to 50 (younger middle-age adults), 51 to 65 (older middle-age), 66 to 80 (older adults), 81+ (very old adults).

#### Analysis and hypotheses

Our purposes were descriptive, and the analysis focused on counts or percentages that described the numbers and proportions of fractures by type disaggregated by gender, age groups, associated injuries, and hospitalizations. The age groups were selected to represent anticipated different health and fracture risk profiles, with young adults having the best bone health and being more likely to engage in higher fracture risk activities. Younger middle-aged adults were considered as being transitional in terms of activity and bone health, while older middle-age individuals (51–65 years) are those with emerging fracture risks. Older adults were considered more likely to have associated comorbidities, and the oldest group (81+) was thought likely to have a higher probability of frailty.

We expected that young adults would be most susceptible to higher trauma “misfortune” fractures, whereas the older middle-aged cohort would contain both active trauma and bone compromised individuals. We expected gender differences in young adults to reflect higher risk-taking or greater participation in high-energy activities in young males. We expected sex differences in bone health to emerge by the 50-year-old age category and to be more pronounced in fractures that are considered fragility fractures such as DRF and PHF. We expected that frailty fractures would be prevalent in the old and very old subgroups. We also expected the longer lifespan of females to be reflected in an excess female with fractures in the oldest category.

We described the age and sex specific counts, percentages and standardized mean differences for specific fractures and tested for sex differences. A significant ANOVA for group comparisons (sex/age) and a standardized mean difference of at least 10% were both needed to be present for us to designate findings as meaningful differences, given our large sample size where even trivial differences would easily reach significant *p*-values.

## Results

### General description of cohort

**Fig. 1:**
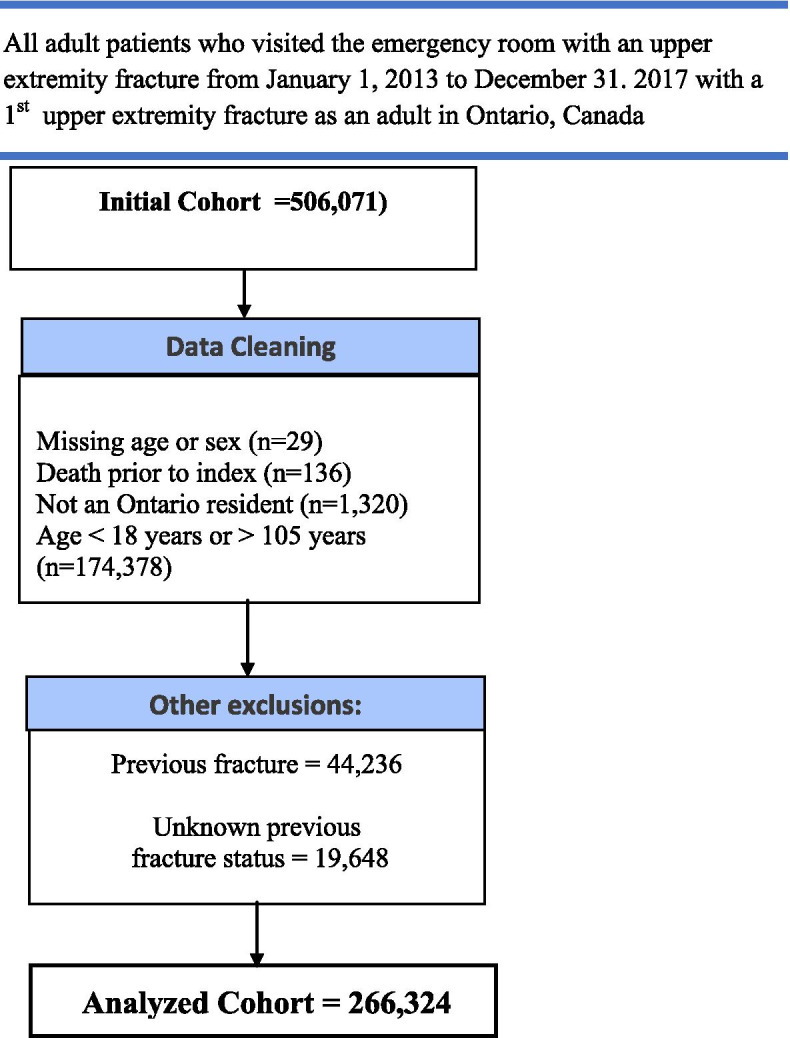
Cohort diagram

After exclusions from the potential cohort (*n* = 506,071), mostly related to age (*n* = 174,378), we were left with a cohort of 266,324 adults with a first upper extremity fracture occurring over a four- year interval (*n* = 44,236 were excluded because they had prior fractures) (Fig. 1). The mean age of the cohort was 51.5 years and was slightly female dominant (51.5%), with demographics of the major fractures and entire cohort in Table 1. Most of the patients were classified as coming from urban settings (81.9%; rural 16.2%), Table 1. The only fracture where the proportion of rurality exceeded 20% was for distal phalangeal fractures (21.1% rural).

**Table 1:** Demographics of the sample and specific upper extremity fractures

Demographics								
Fracture Type	n	Age (years)			Sex (%)		Location (%)*	
		Mean (SD)	Median	IQR	Women	Men	Urban	Rural
**All 1**^**st**^ **incident upper extremity fractures**								
**Entire cohort**	266,324	52 (21)	53	33-67	51.5	48.5	82	17
**Fractures by Region**								
**Shoulder**	48,582	61 (20)	63	49-77	56	43	81	17
**Elbow**	35,362	52 (20)	54	36-67	60	40	85	14
**Wrist**	80,010	55 (20)	57	41-70	65	35	83	16
**Hand**	93,673	42 (18)	40	26-55	33	66	80	18
**Hand and Wrist**	5967	54 (20)	56	37-70	59	41	82	15
**Multiple UE regions**	8697	56 (21)	57	39-72	**57**	43	79	19
**Specific Fractures**								
**Clavicle**	15,121	51 (22)	50	31-66	34	**66**	80	18
**Scapula**	3255	57(20)	57	43-72	33	67	79	20
**Proximal Humerus**	21,765	68(16)	69	59-81	74	**26**	83	**16**
**Humerus Shaft**	2396	**61(20)**	**63**	49-76	**61**	39	82	17
**Humerus, Unspecified**	1992	65 (18)	66	55-78	68	**32**	84	14
**Shoulder, Multiple**	4053	63(19)	65	52-79	57	**43**	79	19
**Distal Humerus**	2738	60(22)	63	44-79	62	**38**	84	15
**Olecranon**	3707	58(20)	59	44-75	**52**	48	85	14
**Coronoid**	714	47(16)	48	34-59	**46**	54	86	13
**Monteggia**	50	46 (19)	48	29-61	**50**	50	74	24
**Radial Head**	13,934	46(18)	47	31-59	60	40	86	13
**Multiple Radius and Ulna**	11,844	57(20)	58	44-72	66	34	84	14
**Ulnar Shaft**	1605	49(21)	48	31-63	44	56	80	18
**Radius Shaft**	449	48 (19)	49	32-62	48	52	82	16
**Ulna and Radial Shaft**	321	46(22)	42	27-63	53	47	86	13
**Distal Radius**	47,407	59(18)	60	49-72	73	27	83	16
**Forearm, Multiple**	8372	55 (20)	56	39-69	62	38	78	20
**Scaphoid**	13,279	44 (20)	42	26-59	50	50	86	13
**Carpal, Other**	4985	50 (18)	52	35-63	39	61	86	13
**Metacarpal 1**	2071	42(20)	38	24-56	29	71	83	15
**Metacarpal 2**	30,462	38 (18)	32	23-49	30	71	82	16
**Metacarpal, Multiple**	2166	40(20)	33	23-52	27	73	80	18
**Proximal Phalanx**	3701	45 (19)	44	28-58	39	61	82	17
**Distal Phalanx**	24,407	45 (17)	45	31-57	29	78	18	21
**Phalanx, Other**	26,211	45 (18)	43	29-57	43	57	82	17
**Finger, Multiple**	4655	47 (19)	47	30-60	30	70	75	23

* This data was missing for 1.5 % of the cohort. DRF= distal radius fracture , DP distal phalanx, = MC= metacarpal, M= multiple , R/U= radius and ulna . UE= upper extremity. The bolded number indicate where an excess burden is present in males or females with 10% or more difference in proportions when all age groups are combined; or for location where the highest proportions exist for either rural or urban

### Fracture locations, types and seasons

**Fig. 2:**
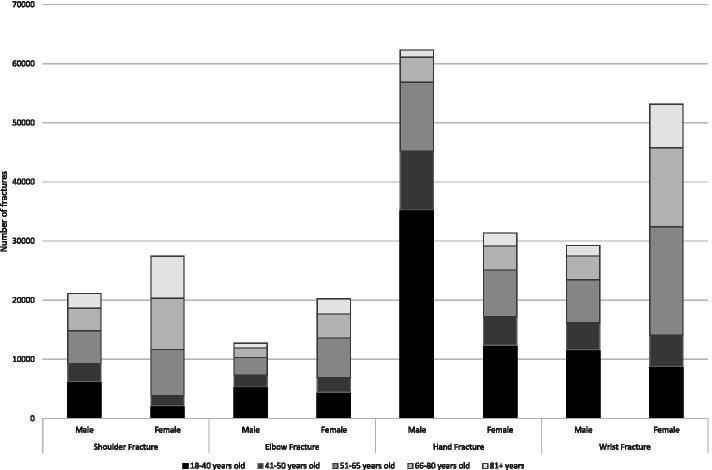
Number of fractures by region by sex by age groups

**Fig. 3:**
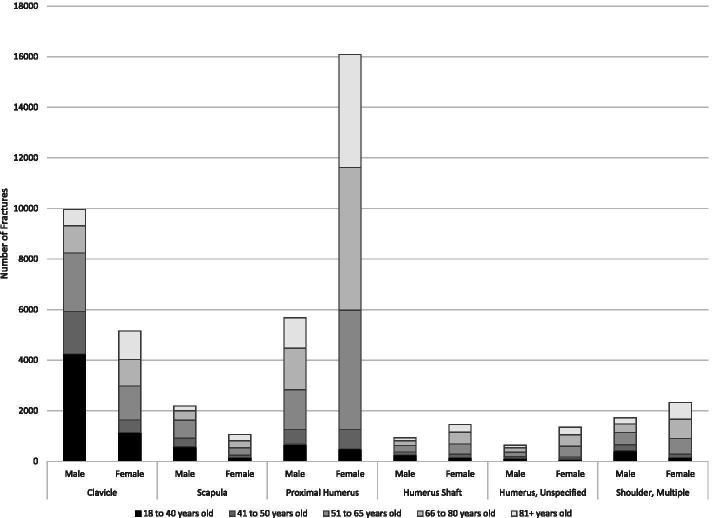
Number of shoulder fractures by type, age and sex. Females=F, Males=M, Clavicle=Clav, Scapula=Scap, Proximal Humerus=PHF, Humerus Shaft=HumS, Humerus, Unspecified=HumU, Shoulder, Multiple=ShldM

**Fig. 4:**
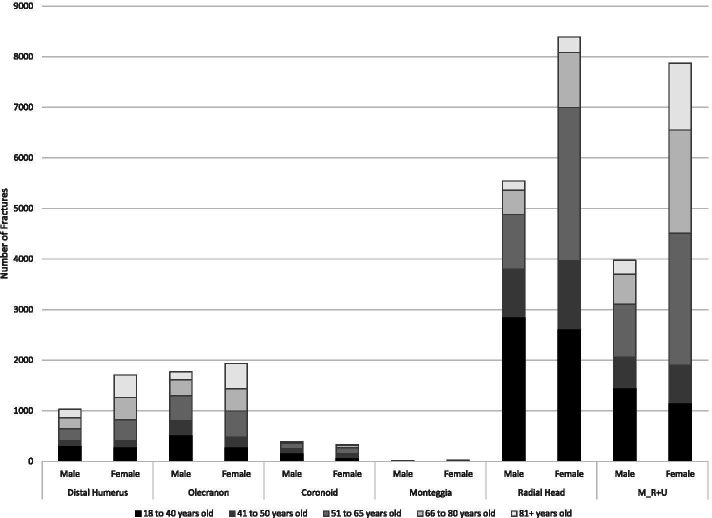
Number elbow region fractures by type, age and sex. Females=F, Males=M, Distal Humerus=DH, Olecranon=Olec,Coronoid=Cor, Monteggia=Mont,Radial Head=RHF

**Fig. 5:**
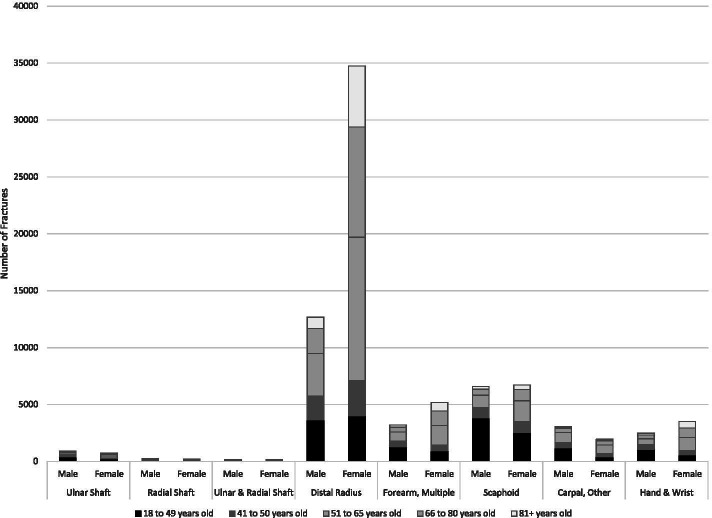
Number of wrist fractures by type, age and sex. Females=F, Males=M, Multiple Radius and Ulna=M_R+U,Ulnar Shaft=UlnS,Radius Shaft=RadS,Ulna and Radial Shaft=Uln+RadS, Distal Radius=DRF, Forearm, Multiple=ForM, Scaphoid=Scapd, Carpal, Other=CarO,Hand & Wrist=H&W

**Fig. 6:**
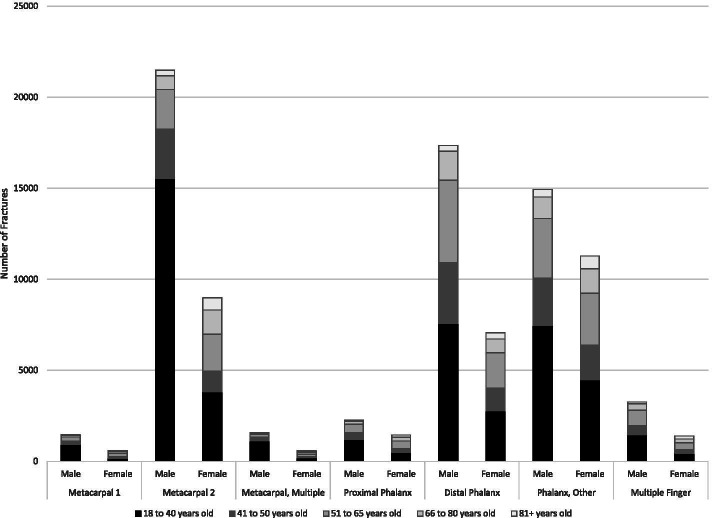
Number of hand fractures by type, age and sex. Females=F, Males=M, metacarpal 1=MC1,Metacarpal 2=MC2, Metacarpal, Multiple=MC_M, Proximal Phalanx=PP, Distal Phalanx=DP,Phalanx, Other=PhaO, Finger, Multiple=Fing_M

Overall, the most common fracture sites were the hand (94 K), followed by the wrist (80 K), shoulder (49 K) and elbow (35 K) – (Table 1; Fig. 2). The most common shoulder fractures were PHF and clavicle (Table 2; Fig. 3). The most common elbow fracture was a radial head fracture (RHF) (Table 2: Fig. 4). DRF represented about one half of the wrist and forearm fractures, with scaphoid being the next most prevalent (Table 2; Fig. 5). Hands had high volumes of fractures of the metacarpals, distal phalanges, and other phalangeal codes (Table 2; Fig. 6). Seasonal findings were mostly unremarkable, likely due to lack of differentiation of seasons in such a large geographic region. Fractures that occurred most in the winter (> 28% occurring between January and March) were DRF, PHF and coronoid fractures (Table 3). Fractures that had a higher predominance in the summer (> 28% occurring between July and September) included: clavicle, scapula, radial head, ulnar or radial shaft (or combined),scaphoid, metacarpal, phalangeal or distal phalangeal fractures and fractures occurring in multiple regions of the upper extremity.

**Table 2:** Fracture characteristics for the cohort, by age and sex

Age	18-40yrs		41-50yrs		51-65yrs		66-80yrs		81+yrs	
Sex	Male	Female	Male	Female	Male	Female	Male	Female	Male	Female
Cohort size	60,078 (67.8%)	28,525 (32.2%)	20,171 (57.8%)	14,749 (42.2%)	28,273 (40.1%)	42,219 (59.9%)	14,089 (31%)	31,419 (69%)	6,577 (24.5%)	20,224 (75.5%)
**Fracture Characteristics, N (%)**										
**Fracture region**										
> 1 region	1,579 (2.6%)	738 (2.6%)	588 (2.9%)	406 (2.8%)	921 (3.3%)	1,487 (3.5%)	437 (3.1%)	1,299 (4.1%)	234 (3.6%)	1,008 (5.0%)
Shoulder	**6,284 (10.5%)**	**2,163 (7.6%)**	**3,039 (15.1%)**	**1,708 (11.6%)**	5,494 (19.4%)	7,805 (18.5%)	3,808 (27.0%)	8,651 (27.5%)	2,510 (38.2%)	7,120 (35.2%)
Elbow	**5,325 (8.9%)**	**4,449 (15.6%)**	**2,018 (10.0%)**	**2,473 (16.8%)**	**2,956 (10.5%)**	**6,696 (15.9%)**	1,633 (11.6%)	4,053 (12.9%)	798 (12.1%)	2,586 (12.8%)
Forearm & Wrist	**11,615 (19.3%)**	**8,811 (30.9%)**	**4,559 (22.6%)**	**5,281 (35.8%)**	**7,288 (25.8%)**	**18,347 (43.5%)**	**4,003 (28.4%)**	**13,363 (42.5%)**	**1,772 (26.9%)**	**7,346 (36.3%)**
Hand	**35,275 (58.7%)**	**12,364 (43.3%)**	**9,967 (49.4%)**	**4,881 (33.1%)**	**11,614 (41.1%)**	**7,884 (18.7%)**	**4,208 (29.9%)**	**4,053 (12.9%)**	**1,263 (19.2%)**	**2,164 (10.7%)**
**Fracture Types**										
Multiple shoulder	418 (0.7%)	148 (0.5%)	244 (1.2%)	131 (0.9%)	480 (1.7%)	630 (1.5%)	339 (2.4%)	755 (2.4%)	247 (3.8%)	661 (3.3%)
Clavicle	**4,241 (7.1%)**	**1,137 (4.0%)**	**1,685 (8.4%)**	**499 (3.4%)**	**2,308 (8.2%)**	**1,339 (3.2%)**	**1,081 (7.7%)**	**1,051 (3.3%)**	**650 (9.9%)**	**1,130 (5.6%)**
Scapula	577 (1.0%)	151 (0.5%)	338 (1.7%)	96 (0.7%)	**711 (2.5%)**	**284 (0.7%)**	**374 (2.7%)**	**286 (0.9%)**	**190 (2.9%)**	**248 (1.2%)**
Proximal Humerus	677 (1.1%)	487 (1.7%)	**586 (2.9%)**	**773 (5.2%)**	**1,569 (5.5%)**	**4,719 (11.2%)**	**1,644 (11.7%)**	**5,639 (17.9%)**	1,203 (18.3%)	4,468 (22.1%)
Humerus, shaft	251 (0.4%)	155 (0.5%)	118 (0.6%)	120 (0.8%)	257 (0.9%)	414 (1.0%)	196 (1.4%)	458 (1.5%)	111 (1.7%)	316 (1.6%)
Humerus, unspecified	120 (0.2%)	85 (0.3%)	68 (0.3%)	89 (0.6%)	169 (0.6%)	419 (1.0%)	174 (1.2%)	462 (1.5%)	109 (1.7%)	297 (1.5%)
Radius Head	**2,855 (4.8%)**	**2,622 (9.2%)**	**940 (4.7%)**	**1,343 (9.1%)**	**1,084 (3.8%)**	**3,031 (7.2%)**	481 (3.4%)	1,090 (3.5%)	184 (2.8%)	304 (1.5%)
Distal Humerus	314 (0.5%)	286 (1.0%)	97 (0.5%)	126 (0.9%)	231 (0.8%)	413 (1.0%)	219 (1.6%)	439 (1.4%)	168 (2.6%)	445 (2.2%)
Olecranon	525 (0.9%)	288 (1.0%)	285 (1.4%)	190 (1.3%)	489 (1.7%)	518 (1.2%)	311 (2.2%)	438 (1.4%)	160 (2.4%)	503 (2.5%)
Coranoid	164 (0.3%)	83 (0.3%)	88 (0.4%)	69 (0.5%)	101 (0.4%)	121 (0.3%)	26 (0.2%)	40 (0.1%)	8 (0.1%)	14 (0.1%)
Monteggia	9 (0.0%)	12 (0.0%)	6 (0.0%)	<=5	8 (0.0%)	8 (0.0%)	<=5	<=5	<=5	<=5
Multiple forearm	1,280 (2.1%)	933 (3.3%)	511 (2.5%)	492 (3.3%)	787 (2.8%)	1,723 (4.1%)	424 (3.0%)	1,278 (4.1%)	201 (3.1%)	743 (3.7%)
Distal Radius	**3,632 (6.0%)**	**4,017 (14.1%)**	**2,086 (10.3%)**	**3,072 (20.8%)**	**3,759 (13.3%)**	**12,615 (29.9%)**	**2,203 (15.6%)**	**9,671 (30.8%)**	**991 (15.1%)**	**5,361 (26.5%)**
Scaphoid	3,840 (6.4%)	2,536 (8.9%)	**877 (4.3%)**	**977 (6.6%)**	1,087 (3.8%)	1,804 (4.3%)	533 (3.8%)	1,005 (3.2%)	**240 (3.6%)**	**380 (1.9%)**
Multiple Radius and Ulna	1,458 (2.4%)	1,158 (4.1%)	**602 (3.0%)**	**744 (5.0%)**	**1,043 (3.7%)**	**2,605 (6.2%)**	**595 (4.2%)**	**2,043 (6.5%)**	**277 (4.2%)**	**1,319 (6.5%)**
Ulnar Shaft	407 (0.7%)	210 (0.7%)	161 (0.8%)	90 (0.6%)	187 (0.7%)	178 (0.4%)	97 (0.7%)	134 (0.4%)	38 (0.6%)	103 (0.5%)
Radius Shaft	110 (0.2%)	50 (0.2%)	46 (0.2%)	36 (0.2%)	45 (0.2%)	64 (0.2%)	20 (0.1%)	43 (0.1%)	12 (0.2%)	23 (0.1%)
Ulna and Radial shaft	90 (0.1%)	64 (0.2%)	20 (0.1%)	17 (0.1%)	22 (0.1%)	30 (0.1%)	13 (0.1%)	38 (0.1%)	6 (0.1%)	21 (0.1%)
Wrist & Hand	1,077 (1.8%)	595 (2.1%)	382 (1.9%)	335 (2.3%)	527 (1.9%)	1,169 (2.8%)	328 (2.3%)	830 (2.6%)	157 (2.4%)	567 (2.8%)
Other carpal	1,179 (2.0%)	406 (1.4%)	476 (2.4%)	262 (1.8%)	874 (3.1%)	764 (1.8%)	**385 (2.7%)**	**364 (1.2%)**	**127 (1.9%)**	**148 (0.7%)**
D1 MC	927 (1.5%)	171 (0.6%)	194 (1.0%)	85 (0.6%)	219 (0.8%)	178 (0.4%)	94 (0.7%)	100 (0.3%)	36 (0.5%)	67 (0.3%)
D2-D5 MC	**15,533 (25.9%)**	**3,819 (13.4%)**	**2,724 (13.5%)**	**1,147 (7.8%)**	**2,160 (7.6%)**	**2,011 (4.8%)**	760 (5.4%)	1,332 (4.2%)	300 (4.6%)	676 (3.3%)
Multiple metacarpal	**1,124 (1.9%)**	**201 (0.7%)**	198 (1.0%)	58 (0.4%)	160 (0.6%)	126 (0.3%)	65 (0.5%)	111 (0.4%)	34 (0.5%)	89 (0.4%)
Proximal Phalanx	<=5	0 (0.0%)	385 (1.9%)	214 (1.5%)	455 (1.6%)	417 (1.0%)	172 (1.2%)	203 (0.6%)	52 (0.8%)	121 (0.6%)
Distal Phalanx	7,580 (12.6%)	2,760 (9.7%)	**3,341 (16.6%)**	**1,266 (8.6%)**	**4,521 (16.0%)**	**1,933 (4.6%)**	**1,580 (11.2%)**	**754 (2.4%)**	**324 (4.9%)**	**348 (1.7%)**
Phalanx, Other	7,470 (12.4%)	4,480 (15.7%)	2,600 (12.9%)	1,910 (13.0%)	**3,267 (11.6%)**	**2,836 (6.7%)**	**1,180 (8.4%)**	**1,346 (4.3%)**	**413 (6.3%)**	**709 (3.5%)**
Multiple Finger	1,448 (2.4%)	444 (1.6%)	525 (2.6%)	201 (1.4%)	**832 (2.9%)**	**383 (0.9%)**	**357 (2.5%)**	**207 (0.7%)**	104 (1.6%)	154 (0.8%)
**Open fracture**	**3,302 (5.5%)**	**669 (2.3%)**	**1,840 (9.1%)**	**398 (2.7%)**	**3,072 (10.9%)**	**821 (1.9%)**	**1,278 (9.1%)**	**540 (1.7%)**	**244 (3.7%)**	**362 (1.8%)**
**Concurrent nerve injury**	190 (0.3%)	62 (0.2%)	107 (0.5%)	34 (0.2%)	180 (0.6%)	59 (0.1%)	74 (0.5%)	30 (0.1%)	14 (0.2%)	16 (0.1%)
Upper arm, major	<=5	0 (0.0%)	<=5	<=5	0 (0.0%)	0 (0.0%)	<=5	0 (0.0%)	0 (0.0%)	0 (0.0%)
Upper arm, minor	17 (0.0%)	16 (0.1%)	7 (0.0%)	6 (0.0%)	9 (0.0%)	14 (0.0%)	<=5	<=5	<=5	<=5
Forearm, major	10 (0.0%)	<=5	<=5	<=5	12 (0.0%)	<=5	6 (0.0%)	<=5	<=5	0 (0.0%)
Forearm, minor	33 (0.1%)	22 (0.1%)	11 (0.1%)	8 (0.1%)	19 (0.1%)	14 (0.0%)	7 (0.0%)	15 (0.0%)	<=5	9 (0.0%)
Hand, major	25 (0.0%)	<=5	14 (0.1%)	<=5	27 (0.1%)	<=5	12 (0.1%)	<=5	<=5	0 (0.0%)
Hand, minor	114 (0.2%)	22 (0.1%)	78 (0.4%)	16 (0.1%)	128 (0.5%)	29 (0.1%)	54 (0.4%)	8 (0.0%)	10 (0.2%)	<=5
**Concurrent tendon injury**	432 (0.7%)	93 (0.3%)	221 (1.1%)	54 (0.4%)	**419 (1.5%)**	**76** **(0.2%)**	**157 (1.1%)**	**28** **(0.1%)**	22 (0.3%)	12 (0.1%)
**Inpatient admission**	2,221 (3.7%)	930 (3.3%)	1,143 (5.7%)	567 (3.8%)	**2,283 (8.1%)**	**2,113 (5.0%)**	1,685 (12.0%)	3,362 (10.7%)	1,355 (20.6%)	4,597 (22.7%)
**Season injured**										
Jan-March	**13,266 (22.1%)**	**7,488 (26.3%)**	**4,669 (23.1%)**	**4,100 (27.8%)**	**6,582 (23.3%)**	**12,146 (28.8%)**	3,402 (24.1%)	7,829 (24.9%)	1,542 (23.4%)	4,631 (22.9%)
April-Jun	15,608 (26.0%)	6,904 (24.2%)	5,020 (24.9%)	3,299 (22.4%)	6,767 (23.9%)	9,411 (22.3%)	3,327 (23.6%)	7,497 (23.9%)	1,551 (23.6%)	5,096 (25.2%)
July-Sept	17,735 (29.5%)	7,828 (27.4%)	5,815 (28.8%)	3,882 (26.3%)	7,953 (28.1%)	10,383 (24.6%)	3,703 (26.3%)	8,062 (25.7%)	1,751 (26.6%)	5,384 (26.6%)
Oct-Dec	13,469 (22.4%)	6,305 (22.1%)	4,667 (23.1%)	3,468 (23.5%)	6,971 (24.7%)	10,279 (24.3%)	3,657 (26.0%)	8,031 (25.6%)	1,733 (26.3%)	5,113 (25.3%)

Cohort size data indicates the numbers/percentages of fractures for each age group by males/females. For each fracture characteristic the numbers/percentages of the cohort are listed as subgroups for each age group and for both males/females. Bolded/shaded cell are those where there are significant sex differences. Small cell numbers <=5 suppressed for confidentiality protection

**Table 3:** Injury characteristics for specific upper extremity fractures

Fracture Characteristics									
Fracture Type	n	% Open	Nerve Injury	Tendon Injury	Hospital Admission	Injury date (%/quarter)			
						Jan- March=5M	April-June	July-Sept	Oct-Dec
Entire cohort	266,324	5	0.3	0.6	8	25	24	27	24
**Fractures by Region**									
**Shoulder**	48582	0.6	0.1	0	17	25	24	27	24
**Elbow**	35362	3	0.3	0.1	11	25	24	**28**	24
**Wrist**	80010	0.8	0.1	0.1	4	**29**	23	25	24
**Hand**	93673	1	0.5	1	1	21	26	**29**	24
**Hand and Wrist**	5967	1	<=5	0.2	1	27	24	26	23
**Multiple UE regions**	4938	7	1	0.4	37	23	24	**29**	24
**Specific Fractures**									
**Clavicle**	15121	0.6	<=5	<=5	12	20	26	**33**	21
**Scapula**	3255	0.5	<=5	<=5	22	24	24	**29**	23
**Proximal Humerus**	21765	**0.3**	0.1	<=5	17	**28**	22	23	**27**
**Humerus Shaft**	2396	1.3	1	<=5	19	**28**	21	25	26
**Humerus, Unspecified**	1992	0	<=5	0	4	**31**	21	22	26
**Shoulder, Multiple**	4053	1.7	0.5	<=5	38	24	24	**27**	24
**Distal Humerus**	2738	4.8	1.5	<=5	27	24	24	**28**	25
**Olecranon**	3707	4.9	0.2	<=5	26	28	21	26	25
**Coronoid**	714	<=5	<=5	<=5	0.4	**31**	23	24	22
**Monteggia**	50	18	0	0	66	20	**32**	24	24
**Radial Head**	13934	<=5	<=5	<=5	2	22	26	**30**	22
**Multiple Radius and Ulna**	11844	4	0.3	0.1	13	27	23	26	24
**Ulnar Shaft**	1605	3	<=5	0.4	6	23	25	**29**	23
**Radius Shaft**	149	4	<=5	<=5	5	23	22	**29**	26
**Ulna and Radial Shaft**	321	16	<=5	<=5	53	27	24	**30**	20
**Distal Radius**	47407	1	0.1	0.1	5	**31**	22	27	23
**Forearm, Multiple**	8372	2	0.2	0.2	9	26	24	27	23
**Scaphoid**	13279	0.2	<=5	0.2	**0.8**	24	25	**28**	23
**Carpal, Other**	4985	0.5	0.1	0.2	1	26	23	27	24
**Metacarpal 1**	2071	3	0.5	1	3	20	27	**30**	22
**Metacarpal 2**	30462	1	0.1	0.3	1	22	25	**28**	24
**Metacarpal, Multiple**	2166	3	0.5	1	3	21	27	28	24
**Proximal Phalanx**	3701	8	0.6	2	2	21	27	29	23
**Distal Phalanx**	24407	23	0.3	1	1	20	26	**29**	25
**Phalanx, Other**	26211	9	0.5	2	1	21	26	**29**	24
**Finger, Multiple**	4655	27	4	1	9	21	25	**29**	25

DRF= distal radius fracture , DP distal phalanx, = MC= metacarpal, M= multiple , R/U= radius and ulna . UE= upper extremity; Bolding is used to highlight where there was some seasonality indicated i.e. the highest proportion of fractures occurring in a specific quarter of the year with a minimum difference of at least 3% over quartiles from other seasons. A (<=5) is used where the numbers were too small for reporting, but not 0 (a measure taken to protect privacy in small cells of health administrative data)

### Description of fracture groups by age/sex profile

Sex and age profiles were quite different across different fracture types (Tables 1 and 2; Figs. 2, 3, 4, 5, 6). Detailed description of age and sex profiles are appended as supplemental figures. The most common fractures were in order: distal radius fractures (DRF), metacarpals, phalangeal fractures, distal phalangeal fractures, proximal humerus fractures, clavicle fractures, radial head fractures and scaphoid fractures. Uncommon fractures included coronoid (*n* = 714), isolated radius shaft (*n* = 449) or combined radial and ulnar shaft (*n* = 321) and Monteggia (*n* = 50).

When examining age profiles by region shoulder fractures had the greatest proportion of the oldest age group and hand fractures had the greatest proportion of the youngest group. (Table 1 and 2) When examining the different demographic profiles of major fracture subtypes, PHF demonstrated the oldest mean age (68 years) and metacarpal fractures (2nd to 4th) the youngest age (38 years). (Table 1). The largest number of fractures occurred in 18 to 40-year-olds for metacarpal, phalangeal, clavicle, radial head, and scaphoid fractures (Table 2; Fig. 2, 3, 4, 5). Clavicle fractures and radial head fractures while presenting in larger numbers for the 18 to 40-year-olds, also had substantial proportions in the 51 to 65-year-old group. For DRF and multiple radial/ulnar fractures, the largest proportion of fractures occurred in the 51–65 group, while in PHF the largest number of fractures number occurred in the 66–80 subgroup. The fractures that were the most female predominant were PHF (73.9%) and DRF (73.3%) (Table 1). The fractures that were most male predominant were distal phalangeal fractures (71.1%) and metacarpal fractures (70.5%) (Table 1). When deconstructing further to create age and gender profiles for specific types of fractures the following descriptive trends (Figs. 2, 3, 4, 5, 6) were observed:

1. Shoulder Fractures (Fig. 3)
  - Scapula fractures -were relatively uncommon with less than 1000 cases per age category. Larger numbers of fractures occurred in males throughout all age groups excepting those over 81 years of age; with a substantial predominance of males up to the age of 65
  - Clavicle Fractures- were the largest volume of fractures in the 18 to 40-year-old category. There was predominance of males with shoulder fractures up to the age of 65, with the sex differential being largest in the youngest age group.
  - Proximal humerus fractures were the largest volume of shoulder fractures in the 66 to 80-year-old age group with more than 8200 fractures. Fracture volumes were low in people under the age of 50. Thereafter, females presented with substantially greater numbers of fractures than males.
  - Humeral shaft fractures-this fracture type was less commonly reported, with less than 700 fractures per age category. There was a larger volume of fractures in males in the 18 to 40-year-old group, with females being more common in the 3 age groups after 50 years of age.
2. Elbow Fractures (Fig. 4)
  - Radial head fractures. The largest number of radial head fractures occurred in the 18 to 40-year-old age group (> 5400 cases) with males being slightly more predominant in presenting cases. In all the subsequent age groups, females were more predominant than males. The largest number of females presenting with radial head fractures occurred in the 51 to 65-year-olds (3031 females versus 1084 males).
  - Olecranon fractures-were a relatively uncommon fracture with less than 1000 cases per age category. The largest volume of these fractures was in the 51 to 65-year-old age group with almost 1000 cases. Prior to 50 years of age males were more predominant in fracture cases, thereafter females were more predominant.
3. Wrist/Forearm (Fig. 5)
  - Distal radius fractures were, by far, the most common wrist fracture, with more than 16,000 cases in the 51 to 65-year-old age group. There were more cases in females at all age groups although the differential was small in the 18 to 40-year-old category and became highly sex differentiated in older age groups, with the 51 to 65-year-old age group (12,615 females versus 3759 males).
  - Scaphoid fractures were most common in 18 to 40-year-olds, with more than 5300 cases. In the youngest group, males were more predominant. In all older age categories females were more prevalent than males.
4. Hand (Fig. 6)
  - Metacarpal fractures of digits 2–5 were most common in the 18 to 40-year-old category and were predominantly males (15,533 males versus 3819 females). Much smaller numbers presented in older categories and the sex differential became trivial by age 51.
  - Proximal /Middle Phalangeal fractures were more common in the 18 to 40-year-old age category (> 13,000 cases) and were male predominant up until the age of 65.
  - Distal phalanx fractures presented in the highest volumes in 18 to 40-year-olds where there were more than 10,000 cases. Males predominated the fracture cases up to the age of 80.
5. Multiple Fractures
  - Fractures in multiple regions of the upper extremity occurred in males more commonly up to the age of 50 with the largest male predominance in the 18 to 40-year-old category (1579 males versus 738 females). After the age of 50 females were more common.
  - Multiple fractures of the shoulder were more common in older age groups. Males were more predominant up to the age of 50 and thereafter females were more common.
  - Multiple fractures of the radius and ulna occurred more commonly in males in the youngest age category, and more commonly in females in all older age groups.

### Associated injury and hospitalizations

Overall, only 4.7% of fractures were coded as open fractures; and nerve (0.3%) and tendon (0.6%) injury was rarely reported (Table 3). A higher proportion of nerve injuries was recorded for fractures in multiple regions (1%) and phalangeal fractures (0.5%). The fracture type that was most recorded as an open fracture was a distal phalangeal fracture (23%). In total 7.6% of the fractures were associated with a hospital admission. Hospital admission rates were highest for fractures occurring in multiple upper extremity regions (37%) and olecranon fractures (26%).

## Discussion

This paper provides a population-level description of first adult upper extremity fractures in a universal healthcare setting. Overall, the hand was the region most affected, reflecting a high volumes of finger fractures. The overall sex/gender ratio of females to males suggests similar rates of fracture (51.5% versus 48.5%), but this overall sex ratio masks large differences in specific age-sex profiles for different fractures. Previous research has typically focused on the most prevalent fractures or has amalgamated many different types of fractures. Our data indicates substantially different age and sex profiles across different upper extremity fracture types. This reinforces the importance of considering sex and lifespan interactions in fracture epidemiology and the loss of information that can arise when amalgamating upper extremity fracture data into too few categories. Since different profiles may be associated with different mechanisms, risks, treatment needs, and outcomes, lumping dissimilar fractures together may mask important findings.

We selected age categories that reflected different bone health stages of the lifespan and found a variety of age-sex fracture patterns. While it is commonly considered that DRF has a bi-modal distribution of young males and older females, we found that females were more prevalent in all age groups, although the difference was small in the 18–40-year-old age group. We saw a large spike in DRF cases in women at 51 years of age, that did not occur for males. In PHF which are typically considered a fragility fracture there was a small excess of males in the 18–40-year-old group; and a large spike in females cases at the 50–65-year-old group. This is consistent with age-related mechanism differences where young males are more likely to incur high trauma or “misfortune” fractures, whereas older females incur a DRF/PHF with low trauma as is typical for a bone-compromised or fragility fracture. This concurs with our mixed methods study of causes of DRF where we examined quantitative data from a cohort of more than 1400 patients [17]. We found that low trauma fractures were more common in those over 45 years of age, and high trauma fractures occurred most often in the youngest (18–24-year-old) group. In the qualitative part of that study, we found the factors contributing to fractures included environment, risk taking behaviors, physical factors, and sports activities. Females have elevated fracture risks due to their bone geometry and composition [18]. At age 50 there is a large increase in DRF and PHF volumes in females compared to males, even though the population contains very similar numbers of males and females at this age [19]. We expected these sex differences due to osteoporosis/osteopenia preferentially affecting females [18] in the middle-aged to older adult females, as this has been previously reported in other epidemiologic studies [3, 5, 7]. An unexpected finding in our current study was that a similar pattern of excess burden in 50+ females in scaphoid fractures, radial head fractures, multiple fractures of the radius and ulna, and humeral shaft fractures, which are not considered fragility fractures. This might mean that our definition of upper extremity fragility fracture may need to be expanded, and that current recurrent fracture prevention strategies which focus on DRF [20] and PHF may be missing opportunities to prevent fractures in at-risk individuals who present with these other upper extremity fractures that share a similar profile. Studies of risk of future spine or hip fractures, and osteoporosis diagnosis following scaphoid fractures, radial head fractures, multiple fractures of the radius and ulna, and humeral shaft fractures are needed to clarify this issue.

Some fractures including scapula, metacarpals, finger fractures and distal phalanx have a high preponderance in young males. This may reflect greater participation in fracture-risk activities/behaviours e.g. contact sports, greater risk-taking, fights/aggressive actions, manual occupations, outdoor work etc. Although we report sex differences because health records measure sex not gender, we acknowledge that there is an interaction between sex and gender. We recognize a lack of non-binary reporting options or clarity on sex/gender in medical records data. To illustrate the potential role of gender we note that metacarpal fractures have a high predominance amongst young males, but this differential disappears in older males. As suggested by the common term “boxers’ fractures” punching behaviours (walls, objects, people) is a common mechanism [21] and our data indicates that younger males are more likely to incur these aggression injuries. However, our data also suggests this aggressive behaviour might dissipate with age, or at least that a first incident decreases with age (since recurrent fractures are not reported in this paper). It is possible that sex hormones and gendered expectations influence aggressive behaviours, especially in young men/males. Conversely, distal phalanx fractures have a substantial predominance in males throughout the lifespan, except the oldest age group. We hypothesize that gender-role differences in occupational and recreational exposures including outdoor work, equipment uses, and manual labour, contribute to lifelong finger fractures risk exposures in males, which would explain why this excess burden is maintained throughout adulthood. While health service data cannot identify why differences occur, these different patterns support hypothesis generation for the source of age sex/gender rate differences that would inform targeted prevention strategies. Quantitative and qualitative studies on fracture mechanisms are needed to explore the explanations for these differences.

In the oldest age group, the number of distal phalanx fractures is higher in females, but this may reflect the greater longevity of females. Further, more older females would be more likely to be living alone which may affect the types of activities they need to do, and the associated risks for fractures [22]. Sex differences in longevity must be considered as a reason why women only become predominant in the oldest age category of fractures. In Canada, at age 80 the ratio of females to males is approximately 4:3, at 85 it is 5:3, at 90 it is 2:1 and at 95 it is 4:1 [19]. Thus, the higher numbers of females having fractures in the oldest category, particularly in fractures that are male predominant in the younger age categories likely reflects the larger volume of females in tshe population. Since we examined fracture volumes and proportions, not rates from the existing population, sex differences in the oldest age group should not be misinterpreted as indicating higher risk. However, the volumes are important since these are an accurate reflection of the burden of first incident fractures that present for management.

Environmental factors are known contributors to fracture [17]. Fractures that occurred more often in the winter months included the classic fragility fractures (DRF, PHF) and olecranon fractures which often occur due to falls from level ground in slippery conditions. Conversely, summer weather is associated with greater participation in outdoor sport and recreational activities which is reflected in a greater burden of clavicle, radial head, fractures in multiple regions of the upper extremity and distal phalangeal fractures. Although only 16% of the fractures overall were classified as rural cases, the fact that the Ontario population is classified as 14% rural, suggests a small excess burden might be attributed to rurality. For example, the largest proportion of cases classified as being rural was for distal phalanx fractures (20%). The nature of both occupational (e.g., fishing and farming) and recreational activities (e.g., off-roading) in rural locations might contribute to increased fracture hazards. These are hypothesized mechanisms since we have no actual data about the circumstances of the fracture including weather or participation in sport, due to the nature of health service data. Further, the demarcation of 10,000 community population is not a good way to differentiate rural life from urban life.

One advantage of administrative data is the potential to study rare diagnoses with large samples. Monteggia fractures are an example, where we hoped to gain novel information on this rare but challenging fracture. Since only 50 cases of Monteggia fractures were reported in our four-year cohort of more than 266,000 cases, longer cohorts would be needed to study the health service profiles of these fractures. We suspect that underreporting may partially explain these low numbers, since not all Monteggia fractures might be identified by that specific code, especially by non-speciality physicians. Potentially, Monteggia were classified as fracture dislocations rather than by their specific name. This may unintentional due be lack of clarity on terminology particularly for non-specialists or be intentional and related to the billing for these 2 codes since fracture -dislocations can have a higher fee. Our data suggests that a coding validation study would be needed prior to conducting any administrative data analyses on this fracture type.

We found very low rates of tendon and nerve injury. The overall rate was influenced by a higher prevalence of nerve injury in finger and distal phalanx fractures, where the nerve injury is unlikely to cause substantial permanent disablement. The relatively higher proportion of 1.1% cases of nerve injury seen in multiple fractures may be of more concern since these would be associated with a higher level of trauma and likely more proximal nerves injuries which has a poorer prognosis. The low rates of reported nerve injury will make it difficult to examine the health service impact of upper extremity nerve injury in future studies unless longer cohort windows are sampled. Tendon injuries were also infrequently reported, and as expected were more common with finger fractures. Since no validation studies have been performed for nerve and tendon codes, we cannot be confident that these estimates are accurate reflections of the prevalence of tendon and nerve injuries associated with upper extremity fractures. It is possible that some nerve and tendon injuries are recognized/coded later when upper extremity specialist surgeons become involved in the case. This should be investigated in a future longitudinal validation study as the inability to accurately identify cases limits the usefulness of health service data for evaluating outcomes of tendon and nerve injury.

We considered that hospital admission might be another indicator of the severity of the injury during which the fracture occurred. This was confirmed since hospital admissions were higher for shoulder and multiple fractures. Some fractures cases may have required hospitalization due to the nature of the surgical reconstruction required e.g., olecranon fractures, Monteggia, or multiple fractures. Others may require hospitalization due to the frailty of the presenting case. Others may have been related to associated injuries. The highest rate of hospitalization occurred when fractures were in multiple regions of the upper extremity which might suggest more complex trauma and associated injuries were important contributing factors to the need for hospitalization.

This study provides a detailed description of first incident upper extremity fractures and suggests that age-sex-specific lifespan patterns vary widely for different fracture types. This may be attributable to sex-specific biologic differences that vary over the lifespan particularly with respect to bone quality, and gender differences in risk-taking behaviours and or gendered work/life roles that also vary across the lifespan. These hypotheses cannot be tested, given the limitations of health service data. However, our data provide more granular definition of the burden of upper extremity fractures than previous studies and indicate that fracture specific profiles are needed in healthcare planning and fracture prevention.

### Strengths and limitations

This study is large and more detailed than previous studies of upper extremity fractures. It discovered age/sex differentiated fracture profiles that have not been previously reported. Despite this, the study has several limitations. Ontario may not represent other regions of Canada or other countries. However, this data represents approximately 40% of the Canadian population and is one of the largest published upper extremity fractures cohort profiles. Since administrative databases were designed for health system management and physician remuneration purposes, there are several inherent limitations to code coverage and validity. Not all fractures would present for care, and some fractures may be missed where imaging is not performed or able to detect early fracture (e.g. scaphoid fractures) or correctly interpreted/coded (e.g. Monteggia fractures). We designated seasons by 3-month intervals to control exposure time, but this does not accurately reflect the weather exposures of interest since Ontario spans over 1 million square km, and the weather patterns vary widely across the province. Imprecision in coding or classification would make it more difficult to describe true differences. Our hypotheses about potential risk factors and behaviours are presumptive since health service data does not contain information about fracture circumstances.

## Conclusions

The demographic profiles of first incident fractures in patients without a prior history of upper extremity fracture as an adult is highly variable across different fracture types. Sex and age greatly influence these profiles. Risk-taking behaviours, sex and gender mechanisms, physiological differences, sex, and aging mechanisms that affect bone quality, seasonal variations/environmental exposures, and social roles/exposures should be explored as contributors to these widely variant fracture profiles. Studies that lumped together all upper extremity fractures may miss important differences in mechanism or prognosis. Future studies should consider the highly variable upper extremity fracture profiles when planning case management and fracture prevention.

## Supplementary Information

**Additional file 1:.**

**Additional file 2:.**

**Additional file 3:.** (PPTX 404 kb)

**Additional file 4:.** (PPTX 145 kb)

## Data Availability

The data set from this study is held securely in coded form at ICES. While data sharing agreements prohibit ICES from making the data set publicly available, access can be granted to those who meet pre-specified criteria for confidential access, available at www.ices.on.ca/DAS.
