# Supplementary material for: Fracture profiles of a 4-year cohort of 266,324 first incident upper extremity fractures from population health data in Ontario
Source: BMC Musculoskelet Disord. 2021 Nov 29;22:996. doi: 10.1186/s12891-021-04849-7 (PMC8630866; doi:10.1186/s12891-021-04849-7)
Supplement: Supplementary file 1 — Additional file 1:. [file 12891_2021_4849_MOESM1_ESM.pdf]

Appendices

Appendix 1. Description of Ontario health administrative databases

Database	Description
Health Services	
Continuing Care Reporting System (CCRS) database	The CCRS database is compiled by the Canadian Institute for Health Information (CIHI) and contains demographic, clinical, functional, and resource utilization information for individuals receiving facility-based continuing care (also known as extended, auxiliary, or complex chronic care) in Ontario hospitals. Clinical assessment data (on the physical, functional, cognitive, and social domains of health) is ascertained using the Resident Assessment Instrument Minimum Data Set (RAI-MDS) version 2.0 which is administered by trained healthcare professionals.
Continuing Care Reporting System Long-Term Care (CCRS-LTC) database	The CCRS-LTC database is compiled by the Canadian Institute for Health Information (CIHI) and comprises all mandatory, clinical assessments performed on nursing home residents in Ontario. In Ontario nursing homes, full assessments are completed on admission, annually, and following any significant change in health status; briefer assessments occur on a quarterly basis. Nursing home assessments are made using the Resident Assessment Instrument Minimum Data Set (RAI-MDS) version 2.0 which are administered by trained healthcare professionals. The instrument captures data on the physical, functional, cognitive, and social domains of health. In a large-scale international study of the reliability of RAI instruments, agreement (average weighted kappa statistics) for the 19 information domains in the long-term care facility instrument ranged from 0.63 to 0.93, with a median value of 0.83.(1)
Discharge Abstract Database (DAD)	The DAD is compiled by the Canadian Institute for Health Information (CIHI) and contains administrative, clinical (diagnoses and procedures/interventions), demographic, and administrative information for all admissions to acute care hospitals in Ontario. At ICES, consecutive DAD records are linked together to form ‘episodes of care’ among the hospitals to which patients have been transferred after their initial admission.<br><br>Prior to April 1, 2002, diagnoses (up to 16 on a given DAD record) are captured using the International Statistical Classification of Diseases, Injuries, and Causes of Death, 9th Revision (ICD-9) coding system and procedures (up to 10 on a given DAD record) are captured using the Canadian Classification of Diagnostic, Therapeutic, and Surgical Procedures (CCP) coding system. Following April 1, 2002, diagnoses (up to 25 on a given DAD record) are captured using the International Statistical Classification of Diseases and Related Health Problems, 10th Revision, Canada (ICD-10-CA) coding system and interventions (up to 20 on a given DAD record) are captured using the Canadian Classification of Health Interventions (CCI) coding system.<br><br>In a hospital medical record reabstraction study of 14,500 hospital discharges from 18 hospital sites between April 2002 and March 2004, DAD records were demonstrated to have excellent agreement (over 99%) for nonmedical information such as demographic and administrative data. Regarding diagnoses,

	median agreement between the original DAD records and the reabstracted records for the 50 most common most responsible diagnoses was 81% (Sensitivity 82%; Specificity 82%).(2) The corresponding median agreement for the 50 most frequently performed surgical procedures was 92% (sensitivity 95%, positive predictive value 91%).
National Ambulatory Care Reporting System (NACRS)	The NACRS is compiled by the Canadian Institute for Health Information (CIHI) and contains administrative, clinical (diagnoses and procedures), demographic, and administrative information for all patient visits made to hospital- and community-based ambulatory care centres (emergency departments, day surgery units, hemodialysis units, and cancer care clinics) in Ontario. At ICES, NACRS records are linked with other data sources (DAD, Ontario Mental Health Reporting System [OMHRS]) to identify transitions to other care settings, such as inpatient acute care or psychiatric care.<br><br>Prior to April 1, 2002, diagnoses (up to 6 on a given NACRS record) are captured using the ICD-9 coding system and procedures (up to 10 on a given NACRS record) are captured using the CCP coding system. Following April 1, 2002, diagnoses (up to 10 on a given NACRS record) are captured using the ICD-10-CA coding system and interventions (up to 10 on a given NACRS record) are captured using the CCI coding system. NACRS emergency department diagnosis codes have been extensively validated.
Ontario Drug Benefit (ODB) program database	The ODB database contains prescription medication claims for those covered under the provincial drug program, mainly: those aged 65 years and older, nursing home residents, patients receiving services under the Ontario Home Care program, those receiving social assistance, and residents eligible for specialized drug programs (e.g., programs which cover the costs of medications for individuals whose medication costs exceed 4% of their net household income and for individuals with rare, serious conditions such as Cystic Fibrosis).<br><br>Each medication claim has an associated prescriber identifier which indicates the health practitioner who wrote the prescription, as well as fields that identify the type and quantity of medication and duration of treatment. A special flag in the ODB database indicates whether the prescription was dispensed to a nursing home resident.<br><br>An audit of 100 randomly selected prescriptions dispensed from 50 Ontario pharmacies determined that the ODB had an error rate of 0.7% and none of the pharmacy characteristics examined (locations, owner affiliation, productivity) were associated with coding errors.(3)
Ontario Health Insurance Plan (OHIP) Claims History Database	The OHIP claims database contains information on inpatient and outpatient services provided to Ontario residents eligible for the province's publicly funded health insurance system by fee-for-service health care practitioners (primarily physicians) and "shadow billings" for those paid through non-fee-for-service payment plans.<br><br>Billing codes on the claims (OHIP fee codes) identify the care

	provider, their area of specialization and the type and location of service. OHIP billing claims also contain a 3-digit diagnosis code - the main reason for the service - captured using a modified version of the ICD, 8th revision coding system. OHIP claims are well completed, but the validity of the diagnosis coding is highly variable.(4)
Ontario Mental Health Reporting System (OMHRS)	The OMHRS is compiled by the Canadian Institute for Health Information (CIHI) and contains administrative, clinical (diagnoses and procedures), demographic, and administrative information for all admissions to adult designated inpatient mental health beds. This includes beds in general hospitals, provincial psychiatric facilities, and specialty psychiatric facilities. Clinical assessment data is ascertained using the Resident Assessment Instrument for Mental Health (RAI-MH), but different amounts of information are collected using this instrument depending on the length of stay in the mental health bed. Multiple assessments may occur during the length of a mental health admission.<br><br>Psychiatric diagnoses are captured using the Diagnostic and Statistical Manual of Mental Disorders, 4th Edition, Text Revision (DSM-IV-TR) coding system. Non-psychiatric diagnoses are captured using the ICD-10-CA coding system.
Same-Day Surgery (SDS) database	The SDS is compiled by the Canadian Institute for Health Information (CIHI) and contains administrative, clinical (diagnoses and procedures), demographic, and administrative information for all patient visits made to day surgery institutions in Ontario.<br><br>Prior to April 1, 2002, diagnoses (up to 16 on a given SDS record) were captured using the ICD-9 coding system and procedures (up to 10 on a given SDS record) were captured using the CCP coding system. Since April 1, 2002, diagnoses (up to 25 on a given SDS record) are captured using the ICD-10-CA coding system and interventions (up to 16 on a given SDS record) are captured using the CCI coding system.
ICES-derived cohorts	
Ontario Asthma Database	The Ontario Asthma Database is created using a definition of ≥2 physician billing claims with a diagnosis of asthma (OHIP diagnosis code: 493) and/or ≥1 inpatient hospitalization or same day surgery record with a diagnosis of asthma (ICD-9 diagnosis code: 493; ICD-10 diagnosis codes: J45, J46; in any diagnostic code space) in a two-year period applied to hospitalization (DAD), same day surgery (SDS), and physician billing claims (OHIP) data to determine the diagnosis date for incident cases of asthma in Ontario.<br><br>For those aged 18 years and older, the above definition, when using primary care chart abstraction as the reference standard, has been demonstrated to have the following performance characteristics: Sensitivity (80.6%), Specificity (81.4%), Positive Predictive Value (72.5%), and Negative Predictive Value (87.3%).(5)
Ontario Congestive Heart Failure (CHF) Database	The Ontario CHF Database is created using a definition of ≥2 physician billing claims with a diagnosis of CHF (OHIP diagnosis code: 428) and/or ≥1 inpatient hospitalization or same day surgery record with a diagnosis of CHF (ICD-9 diagnosis code: 428; ICD-

	10 diagnosis code: I50; in the primary diagnostic code space) in a two-year period applied to hospitalization (DAD), same day surgery (SDS), and physician billing claims (OHIP) data to determine the diagnosis date for incident cases of CHF in Ontario.<br><br>When using electronic medical record data abstraction as the reference standard, the above definition has been demonstrated to have the following performance characteristics: Sensitivity (84.8%), Specificity (97.0%), and Positive Predictive Value (55.3%).(6)
Ontario Chronic Obstructive Pulmonary Disease (COPD) Database	The Ontario COPD Database is created using two separate algorithms applied to inpatient hospitalization (DAD), same day surgery (SDS) records, and physician billing claims (OHIP) data to determine the diagnosis date for incident cases of COPD in Ontario.<br><br>In an algorithm which maximizes sensitivity, the definition for COPD is any physician billing claim with a diagnosis for COPD (OHIP diagnosis codes: 491, 492, 496) or any inpatient hospitalization or same day surgery record with a diagnosis for COPD (ICD-9 diagnosis codes: 491, 492, 496; ICD-10 diagnosis codes: J41- J44; in any diagnostic code space). When using expert panel review of primary care charts as the reference standard, this definition has been shown to have the following performance characteristics: Sensitivity (85.0%), Specificity (78.4%), Positive Predictive Value (57.5%), and Negative Predictive Value (93.8%).(7)<br><br>In an algorithm which maximizes specificity, the definition for COPD is ≥3 physician billing claims with a diagnosis for COPD (OHIP diagnosis codes: 491, 492, 496) or ≥1 inpatient hospitalization or same day surgery record with a diagnosis for COPD (ICD-9 diagnosis codes: 491, 492, 496; ICD-10 diagnosis codes: J41, J42, J43, J44; in any diagnostic code space) in a two-year period. When using expert panel review of primary care charts as the reference standard, this definition has been shown to have the following performance characteristics: Sensitivity (57.5%), Specificity (95.4%), Positive Predictive Value (81.3%), and Negative Predictive Value (86.7%).(7)
ICES Mother-Baby Linked Database (MOMBABY)	The ICES MOMBABY Database links the DAD inpatient admission records of delivering mothers and their newborns. From 2002 onward, this linkage is performed deterministically using a maternal-newborn chart matching number. Prior to 2002, mothers were linked to their children by matching on the institutions they were admitted, their postal codes, and their admission/discharge dates.
Ontario Diabetes Database (ODD)	The ODD is created using algorithms applied to inpatient hospitalization (DAD) records, same day surgery (SDS) records, and physician billing claims (OHIP) data to determine the diagnosis date for incident cases of diabetes in Ontario.<br><br>For adults aged 19 years and greater, the definition for diabetes is 2 physician billing claims with a diagnosis for diabetes (OHIP diagnosis code: 250) or 1 inpatient hospitalization or same day surgery record with a diagnosis for diabetes (ICD-9 diagnosis code: 250; ICD-10 diagnosis codes: E10, E11, E13, E14; in any

	diagnostic code space) within a 2 year period. Physician claims and hospitalizations with a diagnosis of diabetes occurring within 120 prior to and 180 days after a gestational hospitalization record were excluded. When using primary care chart abstraction as the reference standard, this definition has been shown to have the following performance characteristics: Sensitivity (86.1%), Specificity (97.1%), Positive Predictive Value (79.8%), and Negative Predictive Value (98.1%).(8)<br><br>For individuals aged 18 years or less, the definition for diabetes is 4 physician billing claims with a diagnosis of diabetes (OHIP diagnosis code: 250) within a 2 year period. Physician claims during the newborn hospitalization episode were excluded. When using primary care chart abstraction as the reference standard, this definition has been shown to have the following performance characteristics: Sensitivity (82.8%), Specificity (98.9%), Positive Predictive Value (99.4%), and Negative Predictive Value (71.2%).(9)
Ontario Human Immunodeficiency Virus (HIV) Database	The Ontario HIV Database is created using a definition of ≥3 physician billing claims with a diagnosis of HIV (OHIP diagnosis codes: 042, 043, 044) in a three-year period applied to physician billing claims (OHIP) data to determine the diagnosis date for incident cases of HIV in Ontario.<br><br>When using primary care chart abstraction as the reference standard, the above definition has been demonstrated to have the following performance characteristics: Sensitivity (96.2%) and Specificity (99.6%).(10)
Ontario Hypertension Database	The Ontario Hypertension Database is created using a definition of ≥2 physician billing claims with a diagnosis of hypertension (OHIP diagnosis codes: 401-405) and/or ≥1 inpatient hospitalization or same day surgery record with a diagnosis of hypertension (ICD-9 diagnosis codes: 401-405; ICD-10 diagnosis codes: I10-I13, I15; in any diagnostic code space) in a two-year period applied to hospitalization (DAD), same day surgery (SDS), and physician billing claims (OHIP) data to determine the diagnosis date for incident cases of hypertension in Ontario. Physician claims and hospitalizations with a diagnosis of hypertension occurring within 120 prior to and 180 days after a gestational hospitalization record are excluded.<br><br>When using electronic medical record data abstraction as the reference standard, the above definition has been demonstrated to have the following performance characteristics: Sensitivity (72%), Specificity (95%), Positive Predictive Value (87%), and Negative Predictive Value (88%).(11)
Ontario Myocardial Infarction Database (OMID)	The OMID contains records of all inpatient hospital admissions for acute myocardial infarctions (ICD-9 diagnosis code: 410; ICD-10 diagnosis code: I21; in the primary diagnostic code space) in Ontario since 1991. These admissions are ascertained using the DAD and exclude in-hospital events and admissions where there had been a previous discharge for acute myocardial infarction in the previous year. This cohort of patients with acute myocardial infarction hospital admissions is linked with hospitalization (DAD), same day surgery (SDS), and physician billing claims data (OHIP) to create indicators of hospital readmission after discharge

	and receipt of cardiac procedures during and after the initial hospital admission.<br><br>When using a clinical registry of acute coronary syndromes from 58 cardiac care units in Ontario as the reference standard, the above definition has been demonstrated to have the following performance characteristics: Sensitivity (92.8%), Specificity (88.9%), and Positive Predictive Value (88.5%).(12)
Ontario Rheumatoid Arthritis Database (ORAD)	The ORAD is created using a definition of ≥3 physician billing claims, and at least 1 claim billed by a musculoskeletal specialist, with a diagnosis of rheumatoid arthritis (OHIP diagnosis code: 714) and/or ≥1 inpatient hospitalization or same day surgery record with a diagnosis of rheumatoid arthritis (ICD-9 diagnosis code: 714; ICD-10 diagnosis codes: M05, M06; in any diagnostic code space) in a two-year period applied to hospitalization (DAD), same day surgery (SDS), and physician billing claims (OHIP) data to determine the diagnosis date for incident cases of rheumatoid arthritis in Ontario.<br><br>When using rheumatologist-confirmed diagnosis as the reference standard, this definition has been shown to have the following performance characteristics: Sensitivity (97%), Specificity (85%), Positive Predictive Value (76%), and Negative Predictive Value (98%).(13) Using primary care records as the reference standard, the performance statistics were: Sensitivity 78%, Specificity 100%, PPV 78%, and NPV 100%.(14)
Acquired cohorts and registries	
Ontario Cancer Registry (OCR)	The OCR is a computerized database of information on all Ontario residents who have been newly diagnosed with cancer since 1964. All new cases of cancer, expect non-melanoma skin cancer, are registered in the information system which is managed and maintained by Cancer Care Ontario (CCO). Data from multiple sources, including DAD and SDS records from CIHI which include a diagnosis of cancer, paper reports from pathology departments with any mention of cancer, electronic reports from the eight Ontario Regional Cancer Centers and from the Princess Margaret Hospital (the specialized institutions treated cancer patients in Ontario), and electronic reports of all deaths of Ontario residents from the Office of the Registrar General of Ontario based on Ontario Provincial death certificates with cancer as the underlying cause of death are linked to compile incident cases of cancer in Ontario.<br><br>Approximately 95% of all diagnosed cancer cases in Ontario are captured by the OCR.(15) When using a clinical registry of head and neck tumours from a provincial regional cancer centre as the reference standard, there was excellent agreement with the OCR for tumour site (81%) and diagnosis date within 1 month (91.5%).(16)
Care provider and facility data	
ICES Physician Database (IPDB)	The IPDB provides information about all physicians who have practiced in Ontario and is comprised of data contained in the OHIP Claims History Database, the OHIP Corporate Provider Database (CPDB), and the Ontario Physician Human Resource Data Centre (OPHRDC) Database. The database contains information on demographics (age, gender, year of graduation,

	school of graduation); specialty (functional and certified); location of practice; and measures of physician activity (billings and workload data).
Population and demographics	
Client Agency Program Enrollment (CAPE) Database	The CAPE Database is a registry of all patients who have ever been rostered to receive care from a particular physician in Ontario and documents the time period in which a patient was rostered to a specific physician.
Immigration, Refugees, and Citizenship Canada's (IRCC) Permanent Resident Database	The Ontario portion of the IRCC Permanent Resident Database includes immigration application records for people who initially applied to land in Ontario since 1985. The dataset contains permanent residents' demographic information such as country of citizenship, level of education, mother tongue, and landing date. New immigrants who are currently residing in Ontario but originally landed in another province are not captured in this dataset.
Office of the Registrar General (ORGD) Vital Statistics Database	The ORGD Vital Statistics Database contains information on all deaths registered in Ontario starting on January 1, 1990. Information on the causes of death (immediate, antecedent, and underlying) recorded on the death certificate are captured. At ICES, we derive a single cause of death variable based on the underlying cause of death if available and, otherwise, the immediate cause of death using the ICD-9 coding system.
OHIP Registered Persons Database (RPDB)	The OHIP RPDB provides basic demographic information (age, sex, location of residence, date of birth, and date of death for deceased individuals) for those issued an Ontario health insurance number. The RPDB also indicates the time periods for which an individual was eligible to receive publicly funded health insurance benefits and the best known postal code for each registrant on July 1st of each year.

Reference List

(1) Hirdes JP, Ljunggren G, Morris JN, Frijters DH, Finne SH, Gray L et al. Reliability of the interRAI suite of assessment instruments: a 12-country study of an integrated health information system. BMC Health Serv Res 2008; 8:277.

(2) Juurlink D, Preyra C, Croxford R, Chong A, Austin P, Tu J et al. Canadian Institute for Health Information Discharge Abstract Database: A Validation Study. 2006. Toronto, Institute for Clinical Evaluative Sciences.
Ref Type: Report

(3) Levy AR, O'Brien BJ, Sellors C, Grootendorst P, Willison D. Coding accuracy of administrative drug claims in the Ontario Drug Benefit database. Can J Clin Pharmacol 2003; 10(2):67-71.

(4) Williams J, Young W. A summary of the quality of health care administrative databases in Canada. In: Goel V, Williams J, Anderson G, Blackstien-Hirsch P, Fooks C, Naylor C, editors. Patterns of Health Care in Ontario: The ICES Practice Atlas. 2nd Edition. Ottawa: Canadian Medical Association; 1996. 339-346.

(5) Gershon AS, Wang C, Guan J, Vasilevska-Ristovska J, Cicutto L, To T. Identifying patients with physician-diagnosed asthma in health administrative databases. Can Respir J 2009; 16(6):183-188.

(6) Schultz SE, Rothwell DM, Chen Z, Tu K. Identifying cases of congestive heart failure from administrative data: a validation study using primary care patient records. Chronic Dis Inj Can 2013; 33(3):160-166.

(7) Gershon AS, Wang C, Guan J, Vasilevska-Ristovska J, Cicutto L, To T. Identifying individuals with physcian diagnosed COPD in health administrative databases. COPD 2009; 6(5):388-394.

(8) Hux JE, Ivis F, Flintoft V, Bica A. Diabetes in Ontario: determination of prevalence and incidence using a validated administrative data algorithm. Diabetes Care 2002; 25(3):512-516.

(9) Guttmann A, Nakhla M, Henderson M, To T, Daneman D, Cauch-Dudek K et al. Validation of a health administrative data algorithm for assessing the epidemiology of diabetes in Canadian children. Pediatr Diabetes 2010; 11(2):122-128.

(10) Antoniou T, Zagorski B, Loutfy MR, Strike C, Glazier RH. Validation of case-finding algorithms derived from administrative data for identifying adults living with human immunodeficiency virus infection. PLoS One 2011; 6(6):e21748.

(11) Tu K, Campbell NR, Chen ZL, Cauch-Dudek KJ, McAlister FA. Accuracy of administrative databases in identifying patients with hypertension. Open Med 2007; 1(1):e18-e26.

(12) Austin PC, Daly PA, Tu JV. A multicenter study of the coding accuracy of hospital discharge administrative data for patients admitted to cardiac care units in Ontario. Am Heart J 2002; 144(2):290-296.

(13) Widdifield J, Bernatsky S, Paterson JM, Tu K, Ng R, Thorne JC et al. Accuracy of Canadian health administrative databases in identifying patients with rheumatoid arthritis: a validation study using the medical records of rheumatologists. Arthritis Care Res (Hoboken) 2013; 65(10):1582-1591.

(14) Widdifield J, Bombardier C, Bernatsky S, Paterson JM, Green D, Young J et al. An administrative data validation study of the accuracy of algorithms for identifying rheumatoid arthritis: the influence of the reference standard on algorithm performance. BMC Musculoskelet Disord 2014; 15:216.

(15) Robles SC, Marrett LD, Clarke EA, Risch HA. An application of capture-recapture methods to the estimation of completeness of cancer registration. J Clin Epidemiol 1988; 41(5):495-501.

(16) Hall S, Schulze K, Groome P, Mackillop W, Holowaty E. Using cancer registry data for survival studies: the example of the Ontario Cancer Registry. J Clin Epidemiol 2006; 59(1):67-76.
