# Supplementary material for: Fracture profiles of a 4-year cohort of 266,324 first incident upper extremity fractures from population health data in Ontario
Source: BMC Musculoskelet Disord. 2021 Nov 29;22:996. doi: 10.1186/s12891-021-04849-7 (PMC8630866; doi:10.1186/s12891-021-04849-7)
Supplement: Supplementary file 3 — Additional file 3:. (PPTX 404 kb) [file 12891_2021_4849_MOESM3_ESM.pptx]

## Slide 1
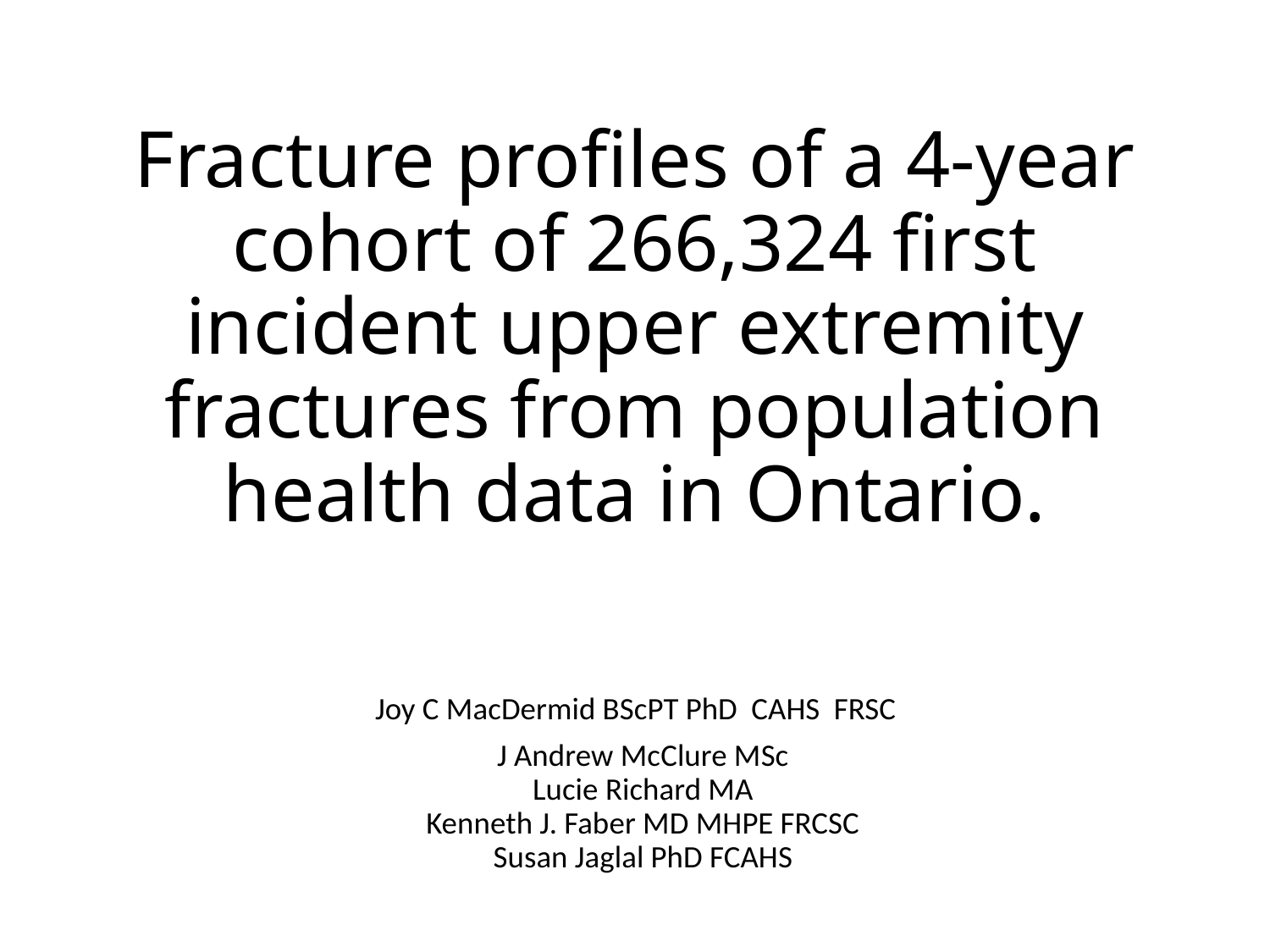

# Fracture profiles of a 4-year cohort of 266,324 first incident upper extremity fractures from population health data in Ontario.
Joy C MacDermid BScPT PhD CAHS FRSC
J Andrew McClure MSc
Lucie Richard MA
Kenneth J. Faber MD MHPE FRCSC
Susan Jaglal PhD FCAHS

## Slide 2
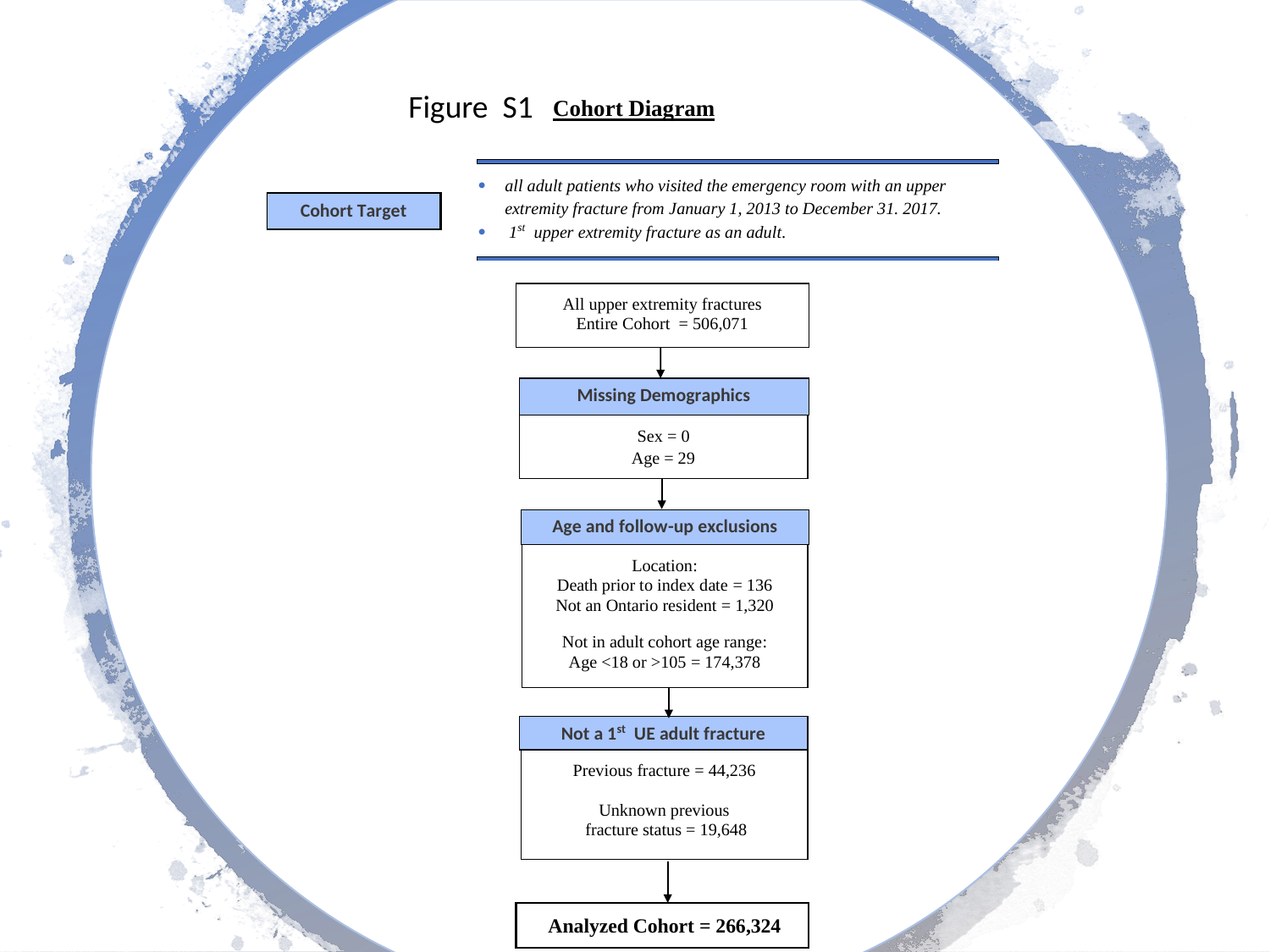

Figure S1

## Slide 3
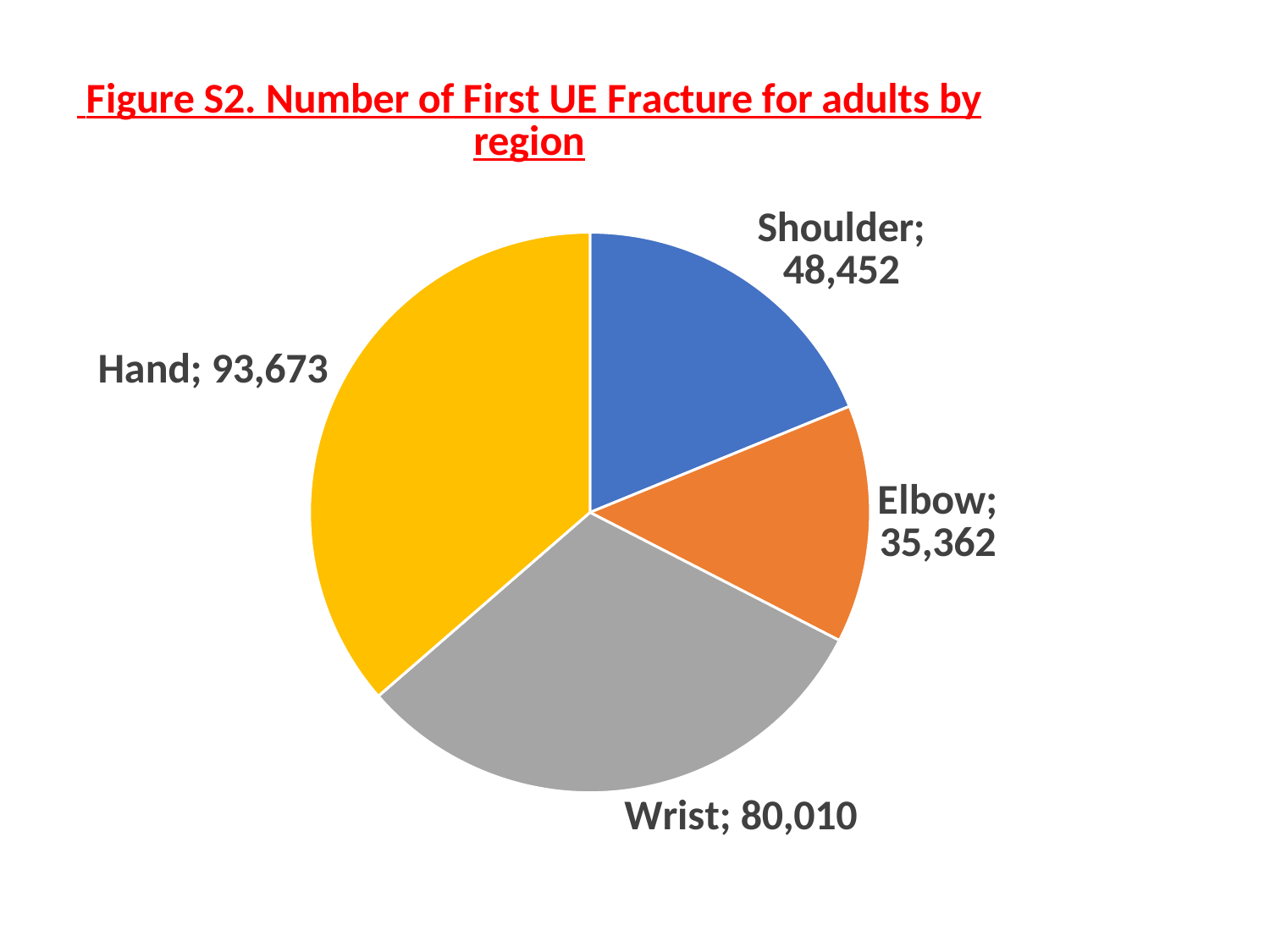

### Chart: Figure S2. Number of First UE Fracture for adults by region
| Category | All UE Fractures |
|---|---|
| Shoulder | 48452.0 |
| Elbow | 35362.0 |
| Wrist | 80010.0 |
| Hand | 93673.0 |

## Slide 4
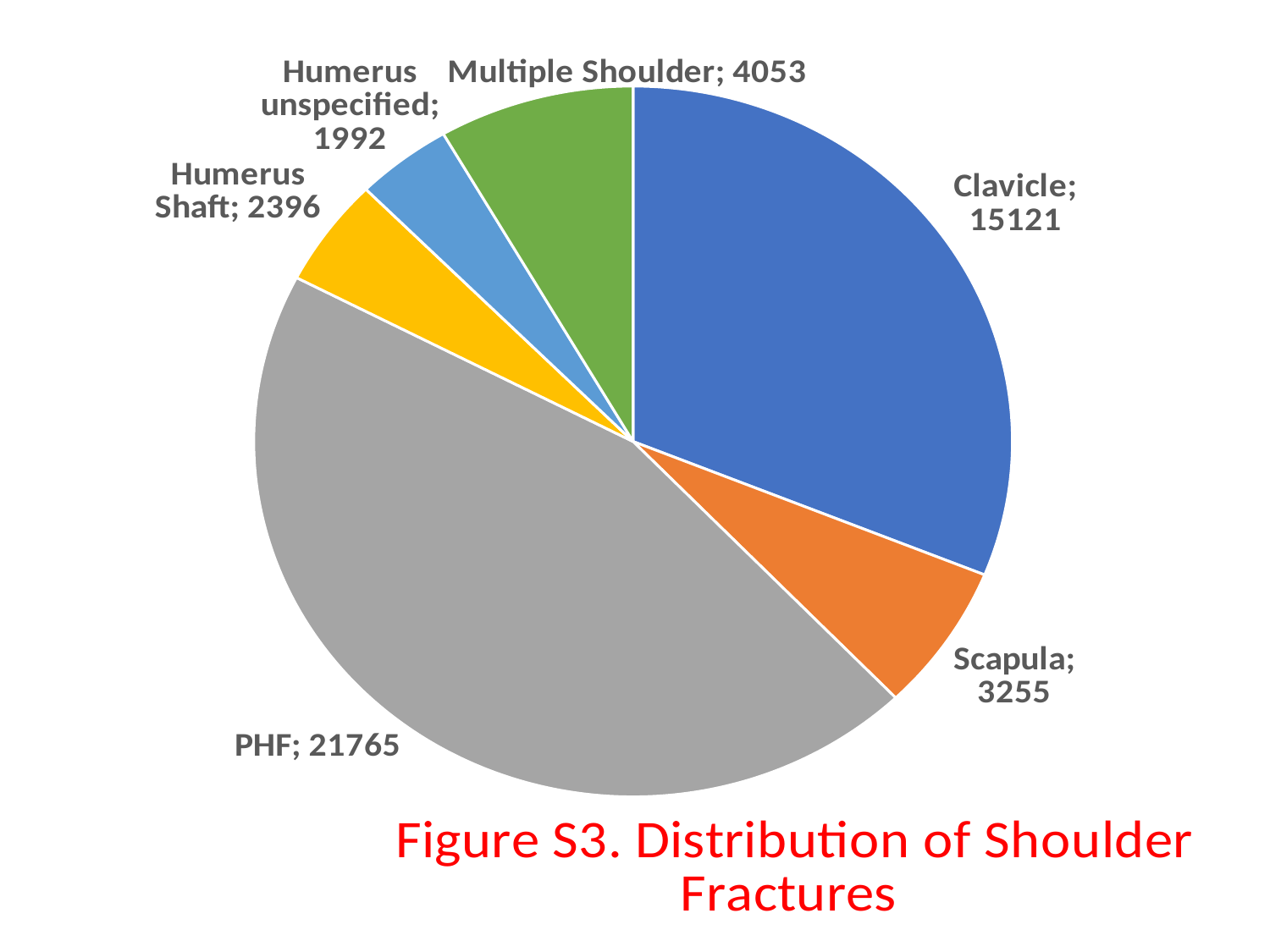

### Chart: Figure S3. Distribution of Shoulder Fractures
| Category | Number of # |
|---|---|
| Clavicle | 15121.0 |
| Scapula | 3255.0 |
| PHF | 21765.0 |
| Humerus Shaft | 2396.0 |
| Humerus unspecified | 1992.0 |
| Multiple Shoulder | 4053.0 |

## Slide 5
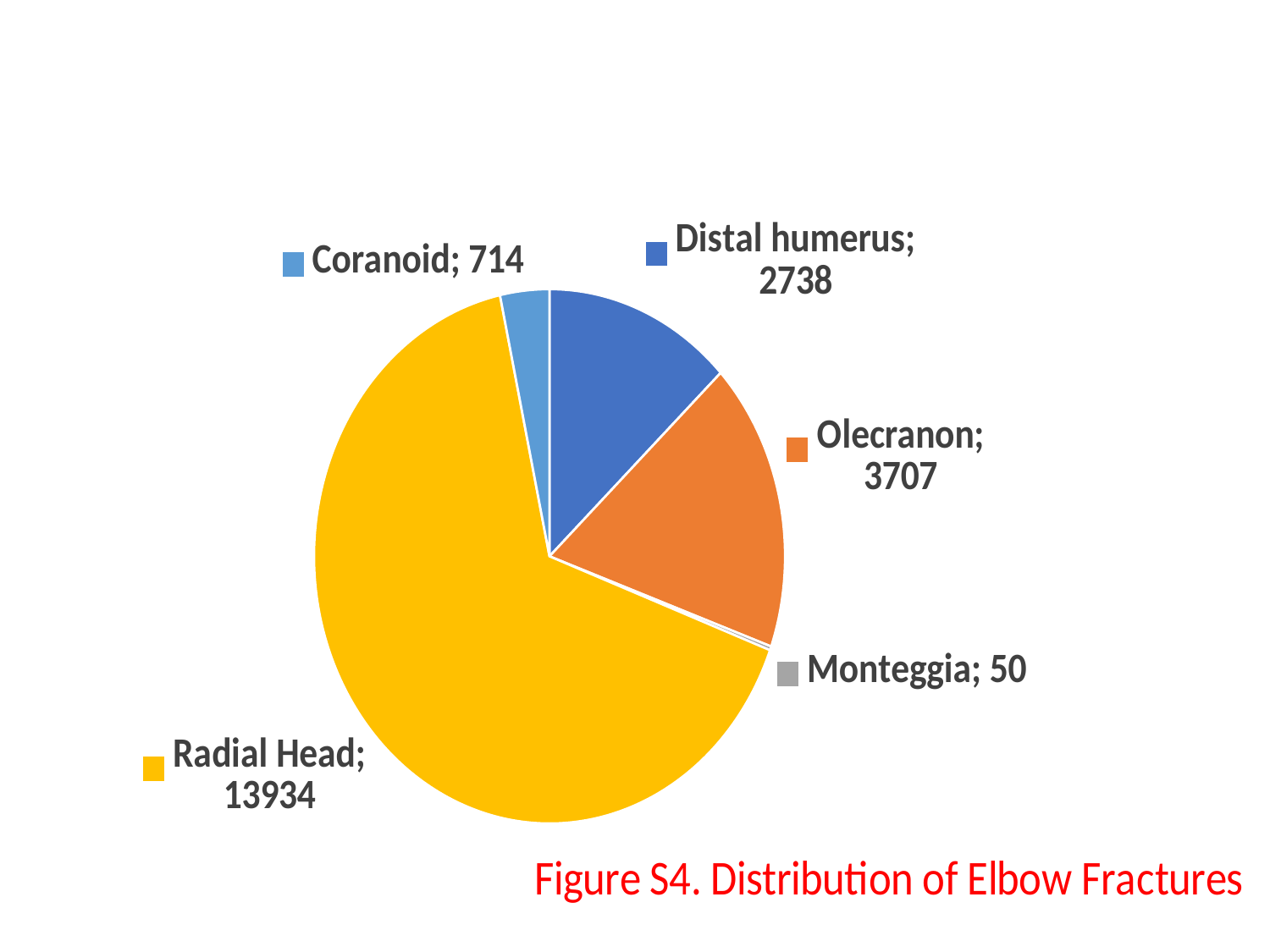

### Chart: Figure S4. Distribution of Elbow Fractures
| Category | Elbow Fractures |
|---|---|
| Distal humerus | 2738.0 |
| Olecranon | 3707.0 |
| Monteggia | 50.0 |
| Radial Head | 13934.0 |
| Coranoid | 714.0 |

## Slide 6
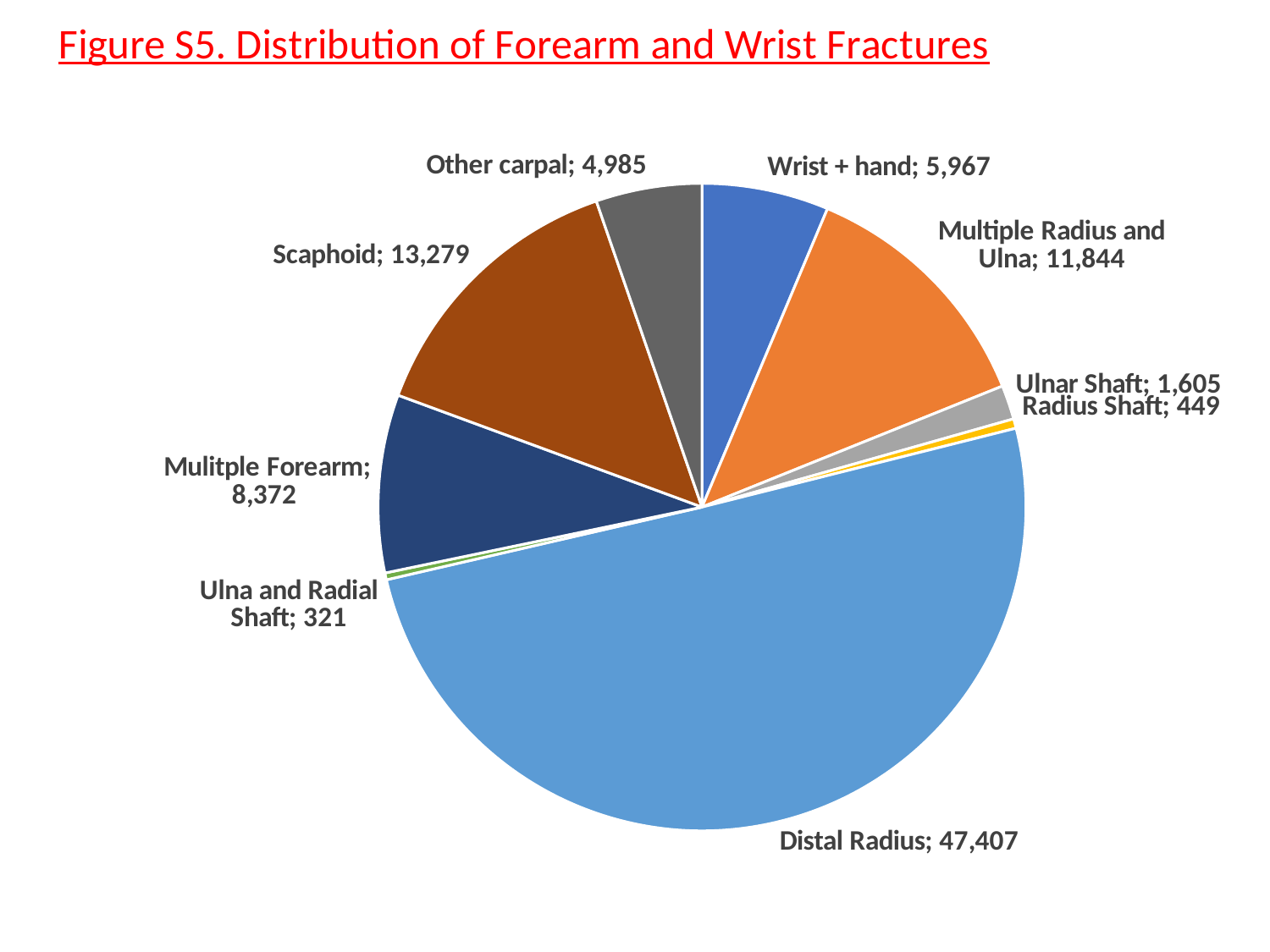

### Chart: Figure S5. Distribution of Forearm and Wrist Fractures
| Category | Forearm and Wrist Fractures |
|---|---|
| Wrist + hand | 5967.0 |
| Multiple Radius and Ulna | 11844.0 |
| Ulnar Shaft | 1605.0 |
| Radius Shaft | 449.0 |
| Distal Radius | 47407.0 |
| Ulna and Radial Shaft | 321.0 |
| Mulitple Forearm | 8372.0 |
| Scaphoid | 13279.0 |
| Other carpal | 4985.0 |

## Slide 7
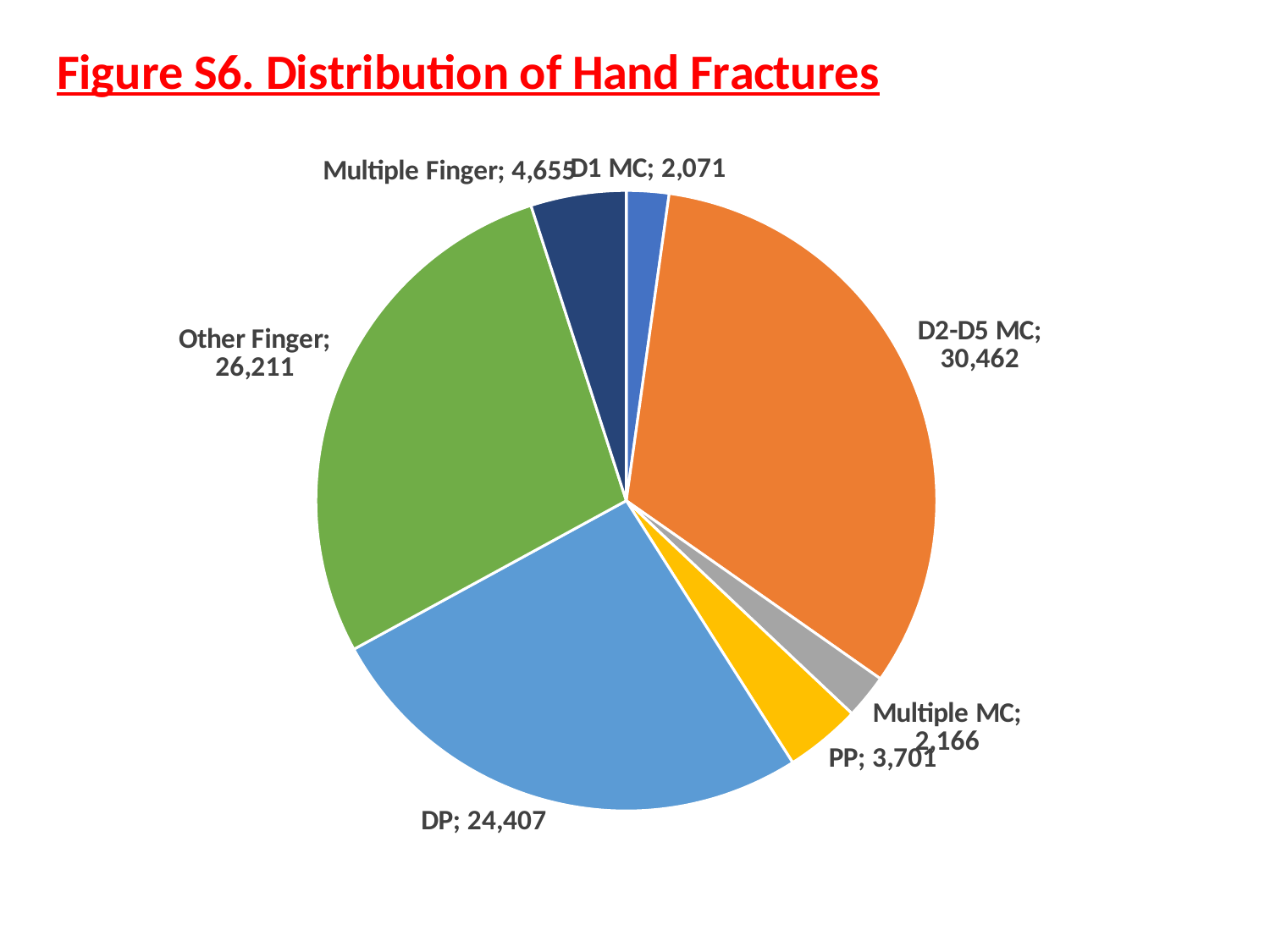

### Chart: Figure S6. Distribution of Hand Fractures
| Category | Hand Fractures |
|---|---|
| D1 MC | 2071.0 |
| D2-D5 MC | 30462.0 |
| Multiple MC | 2166.0 |
| PP | 3701.0 |
| DP | 24407.0 |
| Other Finger | 26211.0 |
| Multiple Finger | 4655.0 |

## Slide 8
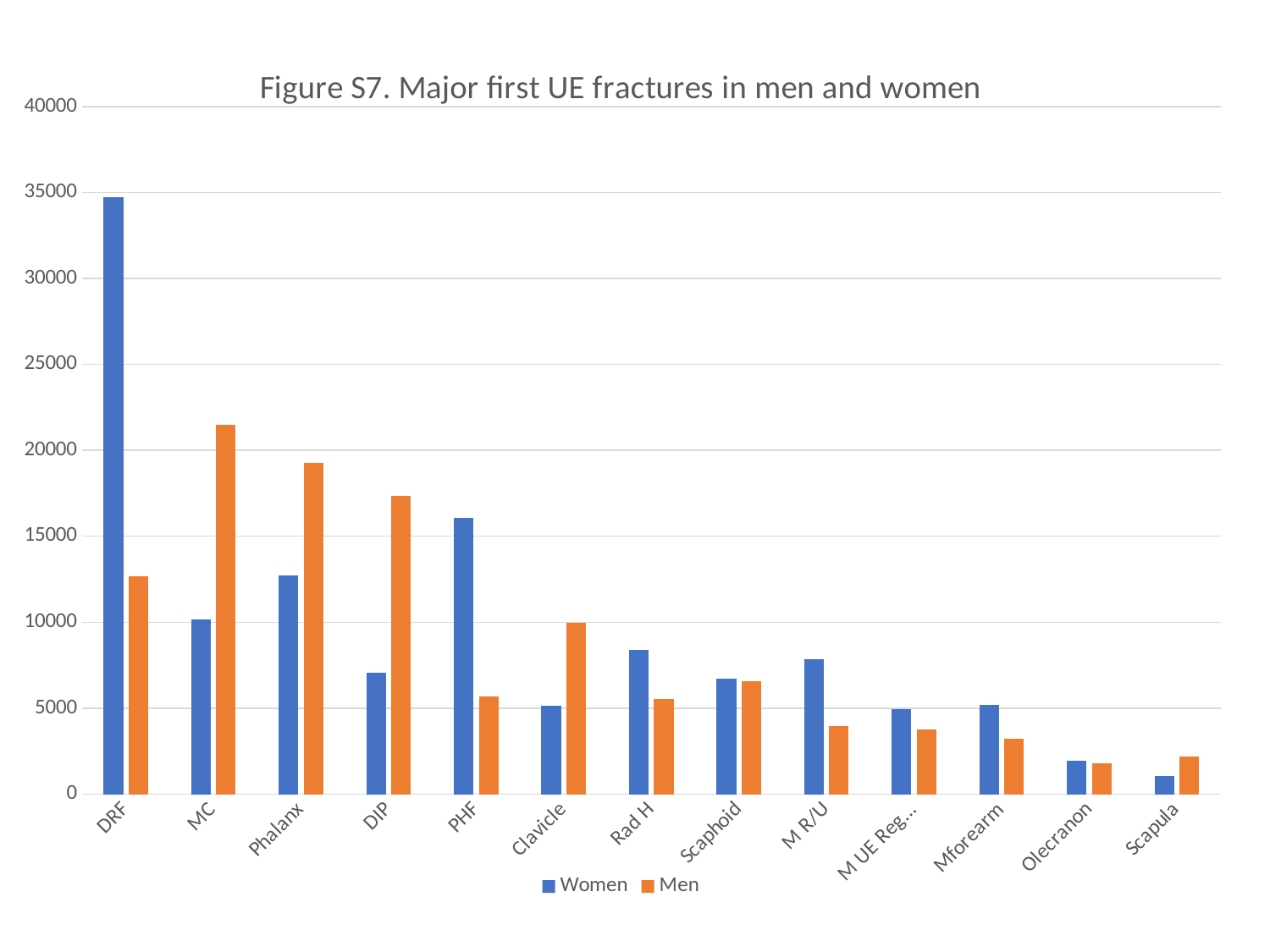

### Chart: Figure S7. Major first UE fractures in men and women
| Category | Women | Men |
|---|---|---|
| DRF | 34736.0 | 12671.0 |
| MC | 10171.0 | 21477.0 |
| Phalanx | 12725.0 | 19287.0 |
| DIP | 7061.0 | 17346.0 |
| PHF | 16086.0 | 5679.0 |
| Clavicle | 5156.0 | 9965.0 |
| Rad H | 8390.0 | 5544.0 |
| Scaphoid | 6702.0 | 6577.0 |
| M R/U | 7869.0 | 3975.0 |
| M UE Regions | 4938.0 | 3759.0 |
| Mforearm | 5169.0 | 3203.0 |
| Olecranon | 1937.0 | 1770.0 |
| Scapula | 1065.0 | 2190.0 |

## Slide 9
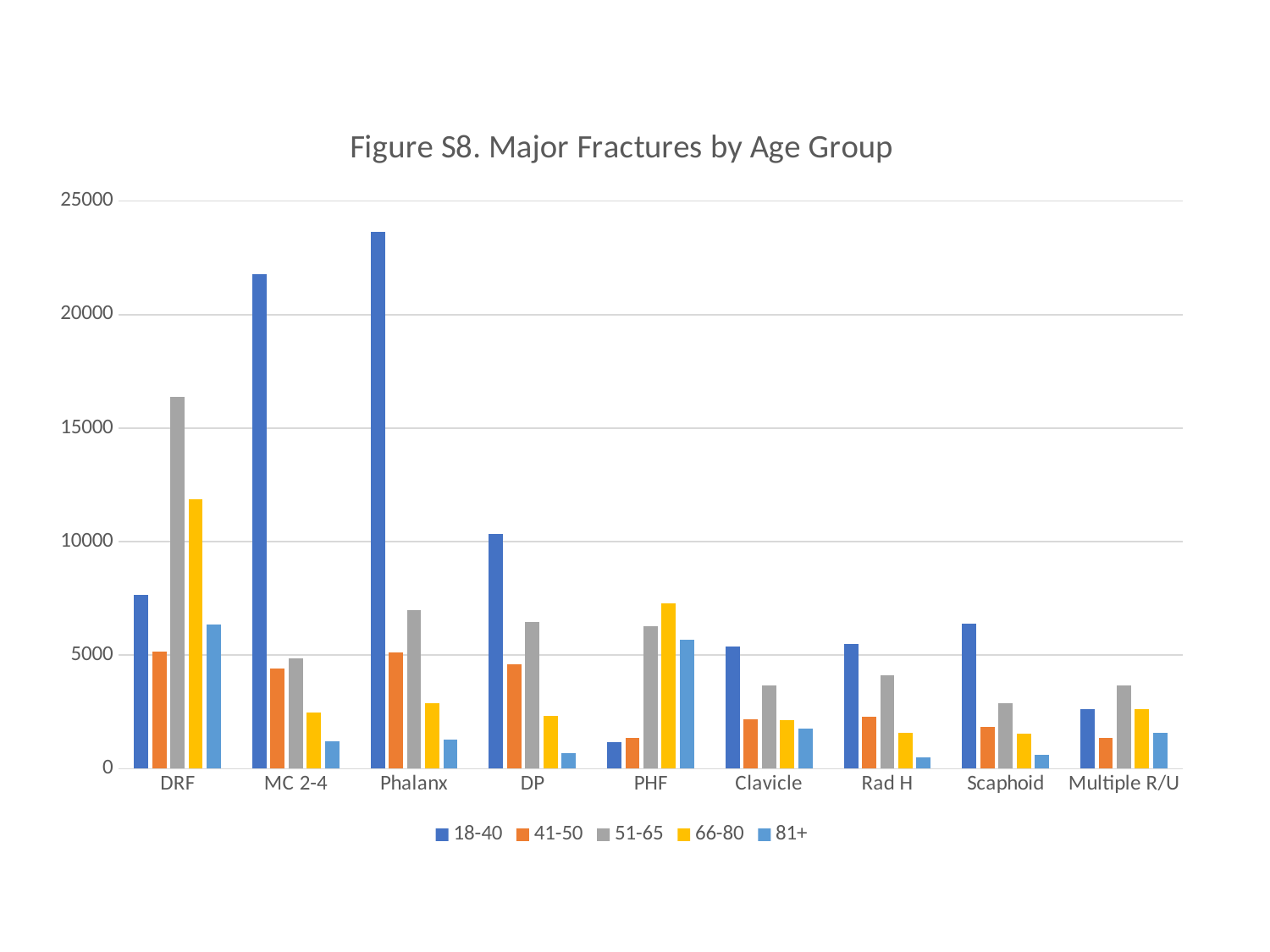

### Chart: Figure S8. Major Fractures by Age Group
| Category | 18-40 | 41-50 | 51-65 | 66-80 | 81+ |
|---|---|---|---|---|---|
| DRF | 7649.0 | 5158.0 | 16374.0 | 11874.0 | 6352.0 |
| MC 2-4 | 21775.0 | 4405.0 | 4854.0 | 2462.0 | 1202.0 |
| Phalanx | 23632.0 | 5109.0 | 6975.0 | 2891.0 | 1295.0 |
| DP | 10340.0 | 4607.0 | 6454.0 | 2334.0 | 672.0 |
| PHF | 1164.0 | 1359.0 | 6288.0 | 7283.0 | 5671.0 |
| Clavicle | 5378.0 | 2184.0 | 3647.0 | 2132.0 | 1780.0 |
| Rad H | 5477.0 | 2283.0 | 4115.0 | 1571.0 | 488.0 |
| Scaphoid | 6376.0 | 1854.0 | 2891.0 | 1538.0 | 620.0 |
| Multiple R/U | 2616.0 | 1346.0 | 3648.0 | 2638.0 | 1596.0 |

## Slide 10
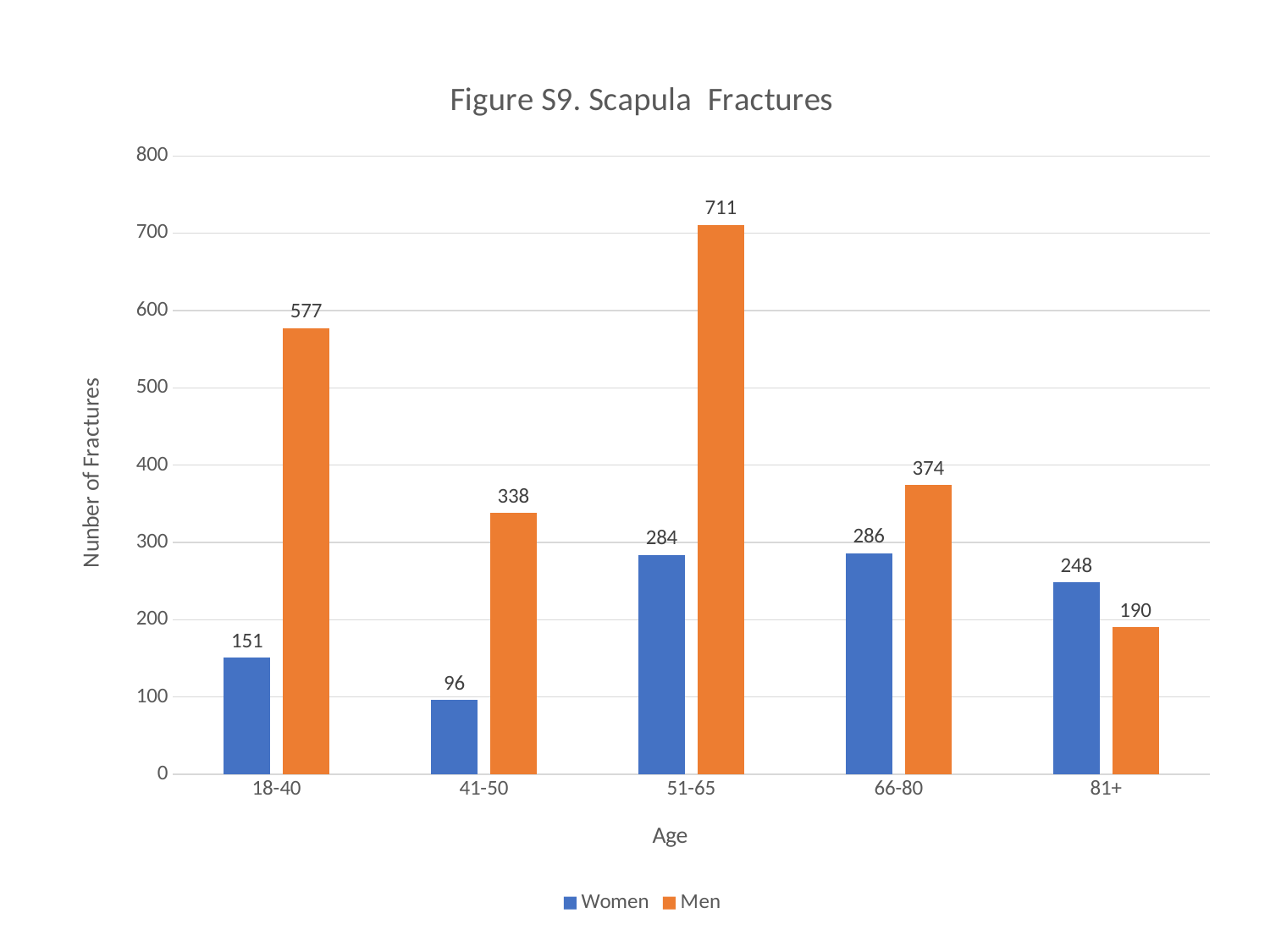

### Chart: Figure S9. Scapula Fractures
| Category | Women | Men |
|---|---|---|
| 18-40 | 151.0 | 577.0 |
| 41-50 | 96.0 | 338.0 |
| 51-65 | 284.0 | 711.0 |
| 66-80 | 286.0 | 374.0 |
| 81+ | 248.0 | 190.0 |

## Slide 11
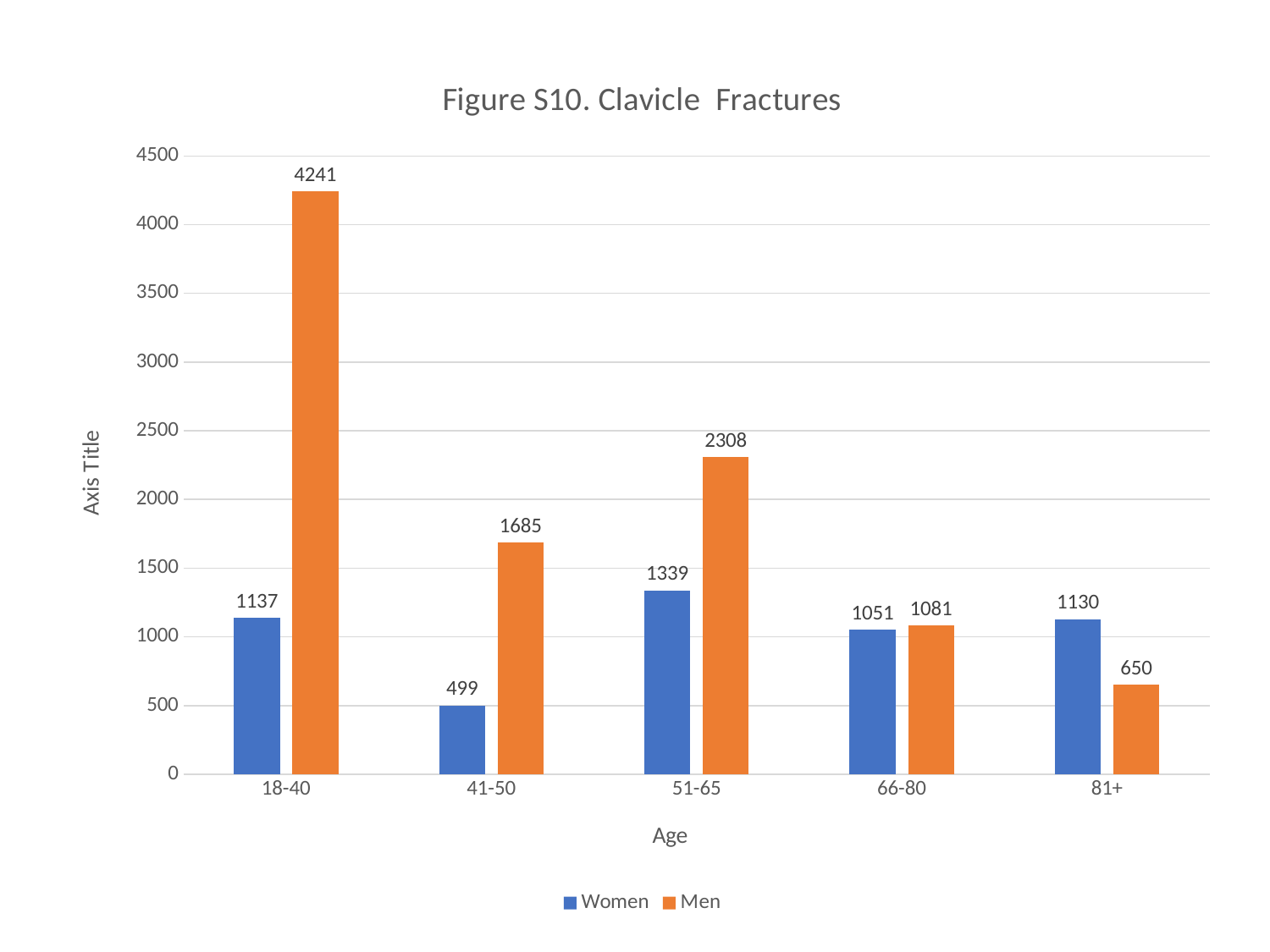

### Chart: Figure S10. Clavicle Fractures
| Category | Women | Men |
|---|---|---|
| 18-40 | 1137.0 | 4241.0 |
| 41-50 | 499.0 | 1685.0 |
| 51-65 | 1339.0 | 2308.0 |
| 66-80 | 1051.0 | 1081.0 |
| 81+ | 1130.0 | 650.0 |

## Slide 12
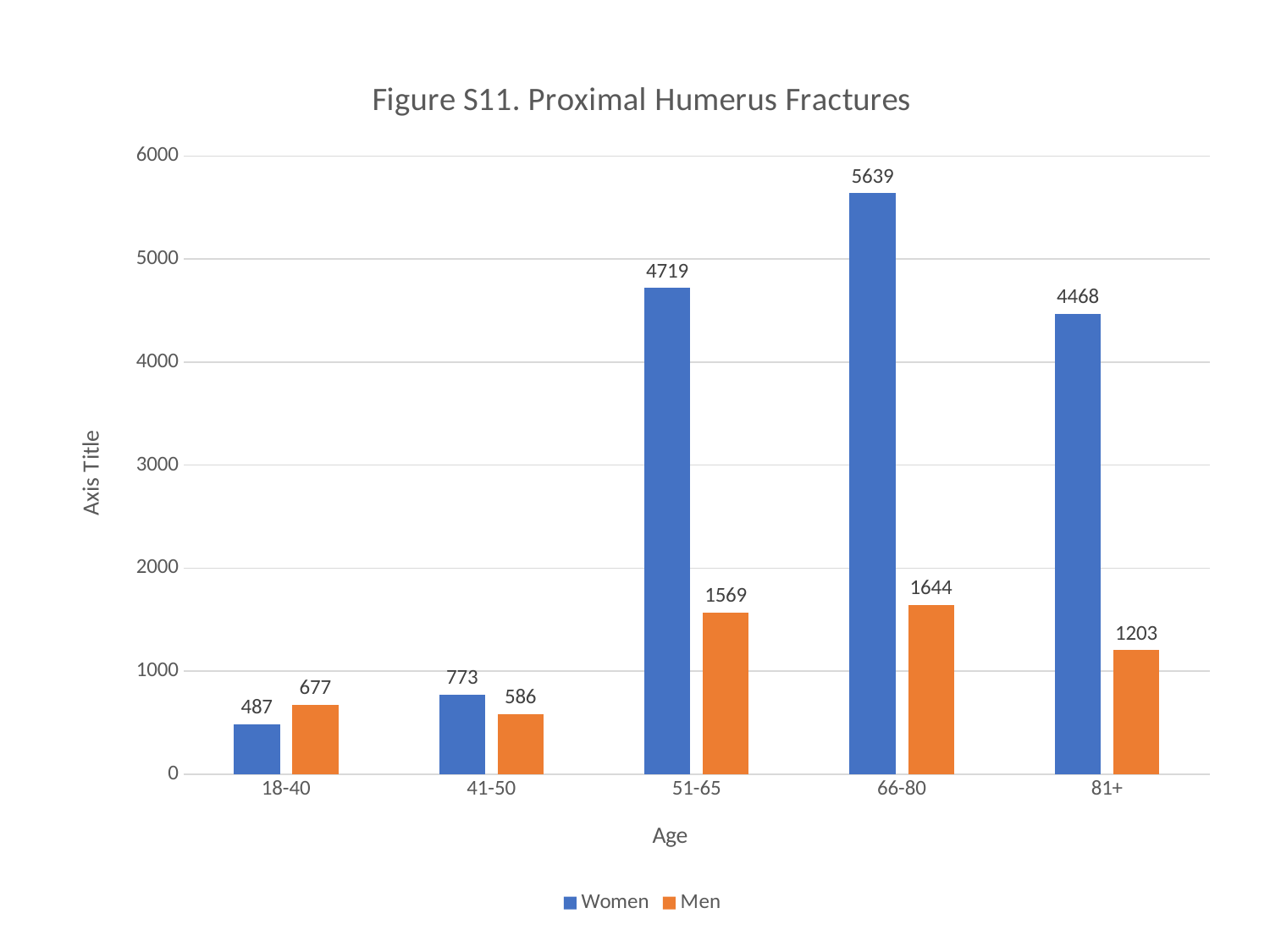

### Chart: Figure S11. Proximal Humerus Fractures
| Category | Women | Men |
|---|---|---|
| 18-40 | 487.0 | 677.0 |
| 41-50 | 773.0 | 586.0 |
| 51-65 | 4719.0 | 1569.0 |
| 66-80 | 5639.0 | 1644.0 |
| 81+ | 4468.0 | 1203.0 |

## Slide 13
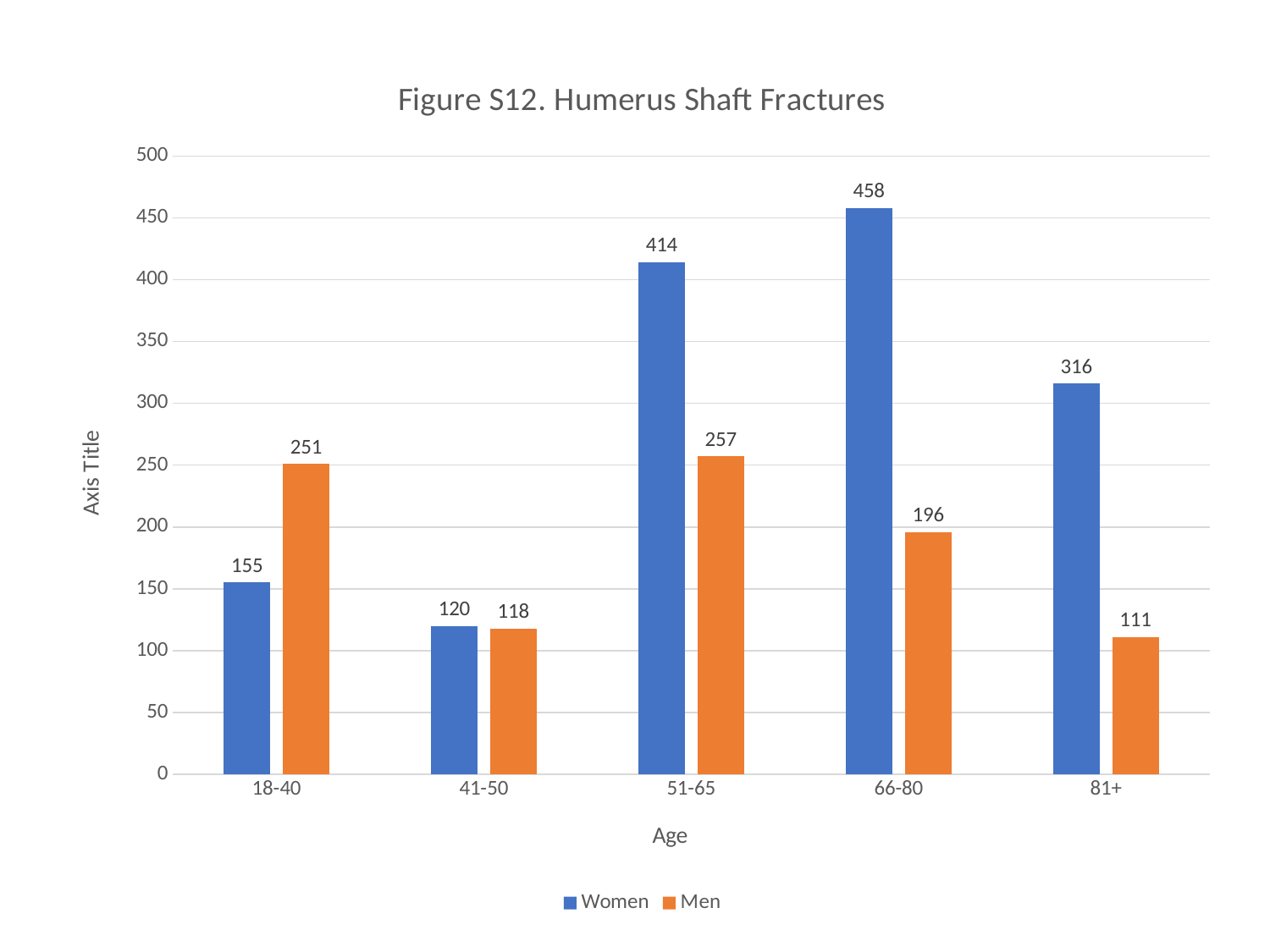

### Chart: Figure S12. Humerus Shaft Fractures
| Category | Women | Men |
|---|---|---|
| 18-40 | 155.0 | 251.0 |
| 41-50 | 120.0 | 118.0 |
| 51-65 | 414.0 | 257.0 |
| 66-80 | 458.0 | 196.0 |
| 81+ | 316.0 | 111.0 |

## Slide 14
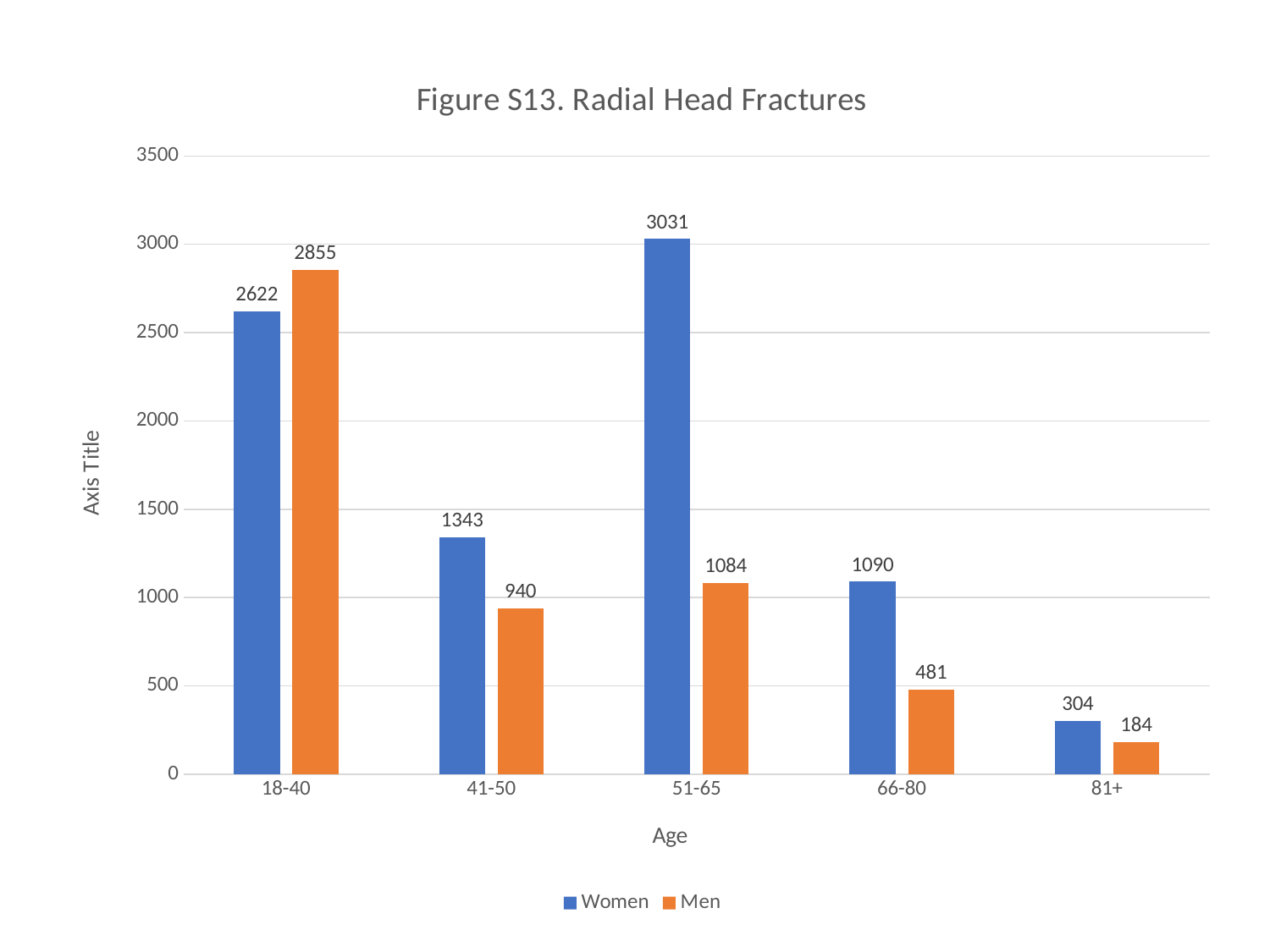

### Chart: Figure S13. Radial Head Fractures
| Category | Women | Men |
|---|---|---|
| 18-40 | 2622.0 | 2855.0 |
| 41-50 | 1343.0 | 940.0 |
| 51-65 | 3031.0 | 1084.0 |
| 66-80 | 1090.0 | 481.0 |
| 81+ | 304.0 | 184.0 |

## Slide 15
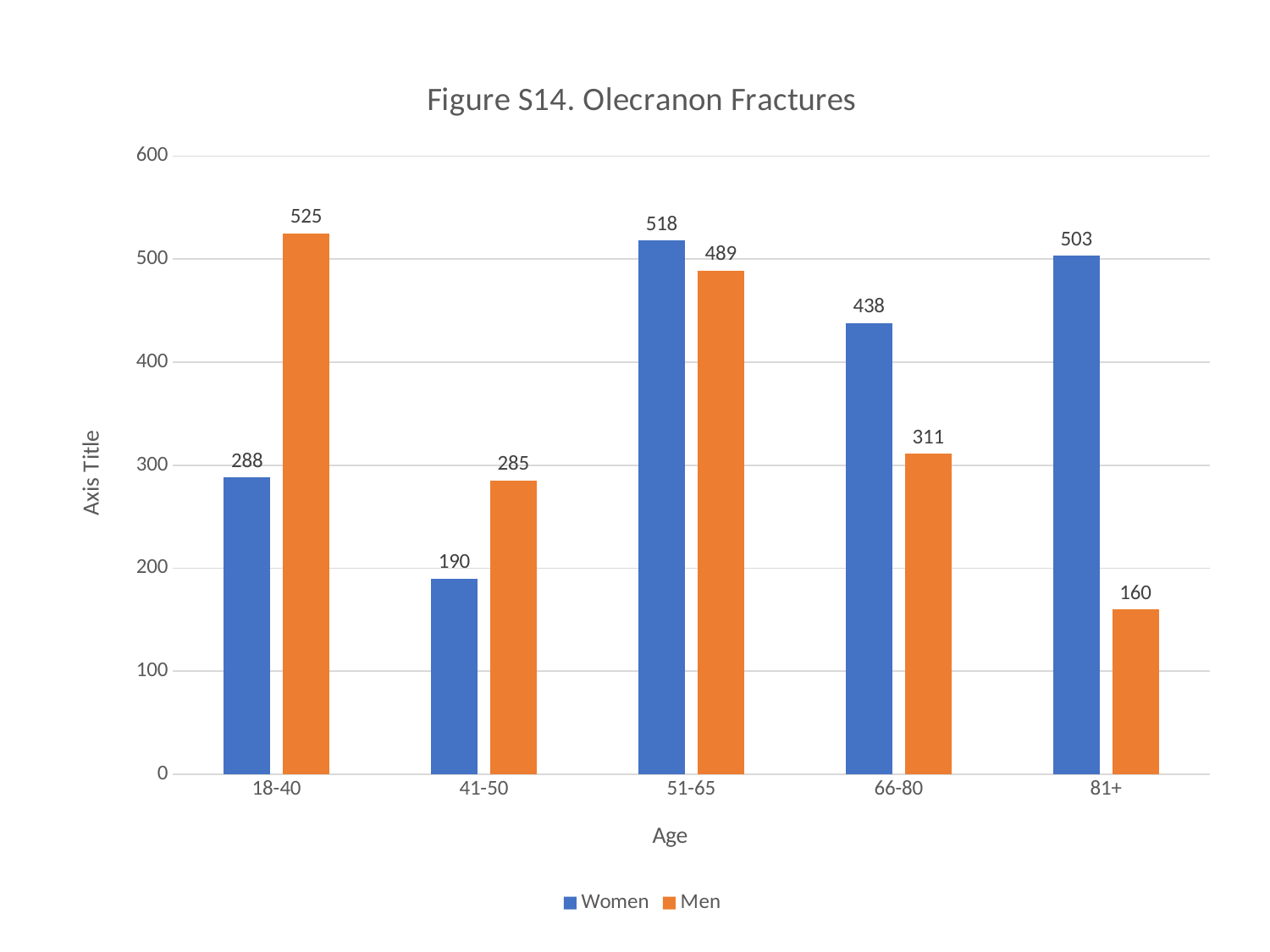

### Chart: Figure S14. Olecranon Fractures
| Category | Women | Men |
|---|---|---|
| 18-40 | 288.0 | 525.0 |
| 41-50 | 190.0 | 285.0 |
| 51-65 | 518.0 | 489.0 |
| 66-80 | 438.0 | 311.0 |
| 81+ | 503.0 | 160.0 |

## Slide 16
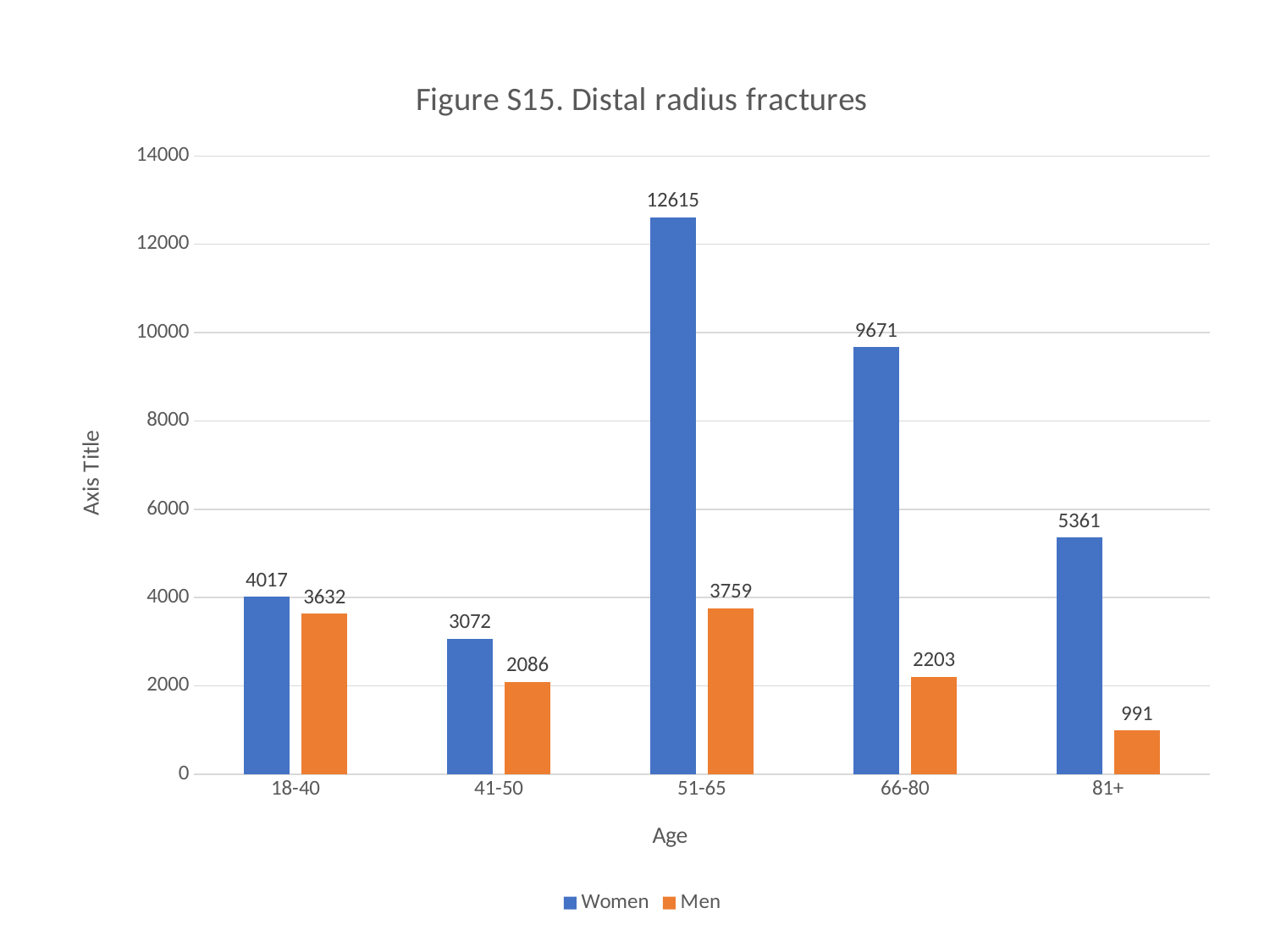

### Chart: Figure S15. Distal radius fractures
| Category | Women | Men |
|---|---|---|
| 18-40 | 4017.0 | 3632.0 |
| 41-50 | 3072.0 | 2086.0 |
| 51-65 | 12615.0 | 3759.0 |
| 66-80 | 9671.0 | 2203.0 |
| 81+ | 5361.0 | 991.0 |

## Slide 17
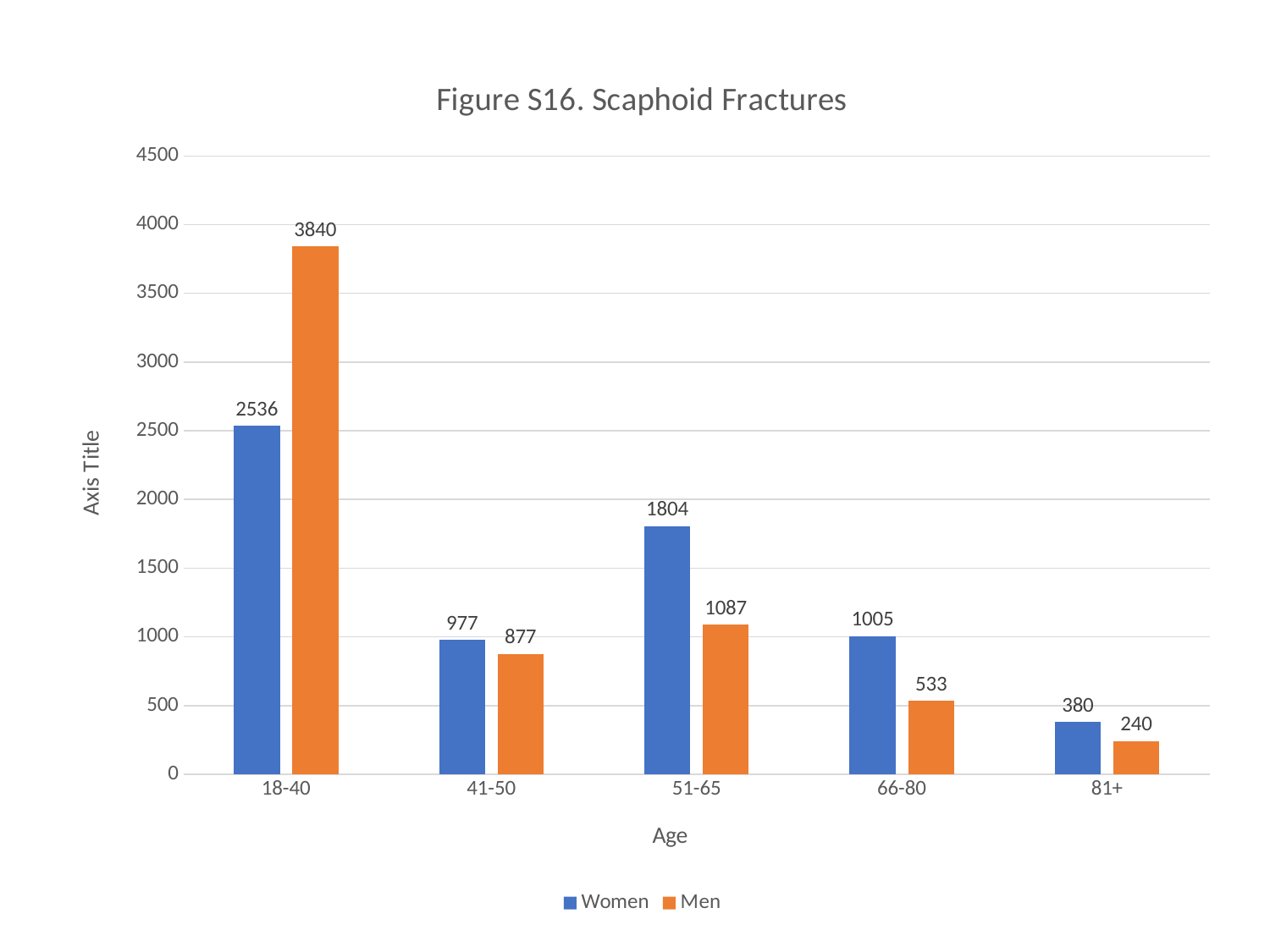

### Chart: Figure S16. Scaphoid Fractures
| Category | Women | Men |
|---|---|---|
| 18-40 | 2536.0 | 3840.0 |
| 41-50 | 977.0 | 877.0 |
| 51-65 | 1804.0 | 1087.0 |
| 66-80 | 1005.0 | 533.0 |
| 81+ | 380.0 | 240.0 |

## Slide 18
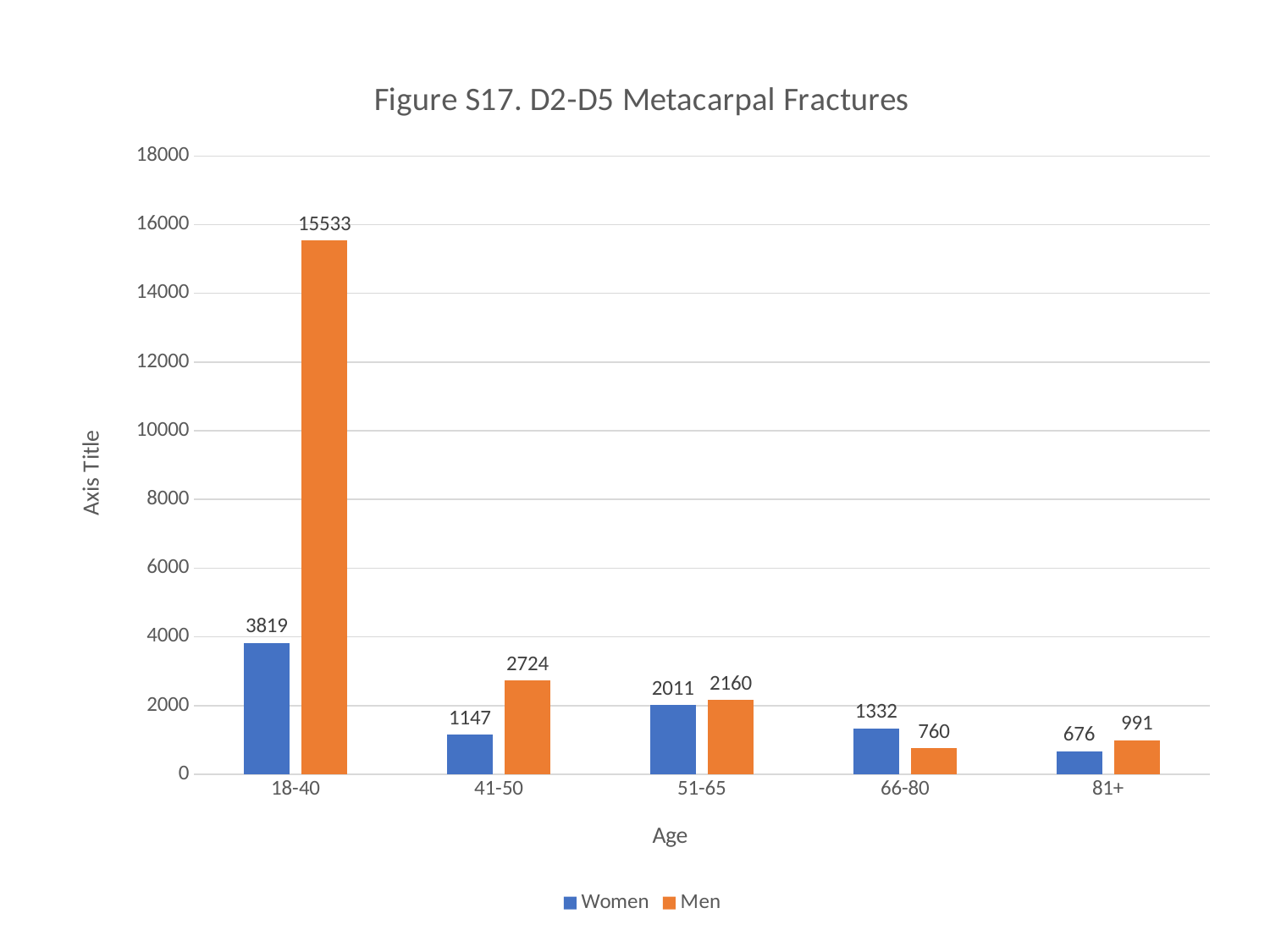

### Chart: Figure S17. D2-D5 Metacarpal Fractures
| Category | Women | Men |
|---|---|---|
| 18-40 | 3819.0 | 15533.0 |
| 41-50 | 1147.0 | 2724.0 |
| 51-65 | 2011.0 | 2160.0 |
| 66-80 | 1332.0 | 760.0 |
| 81+ | 676.0 | 991.0 |

## Slide 19
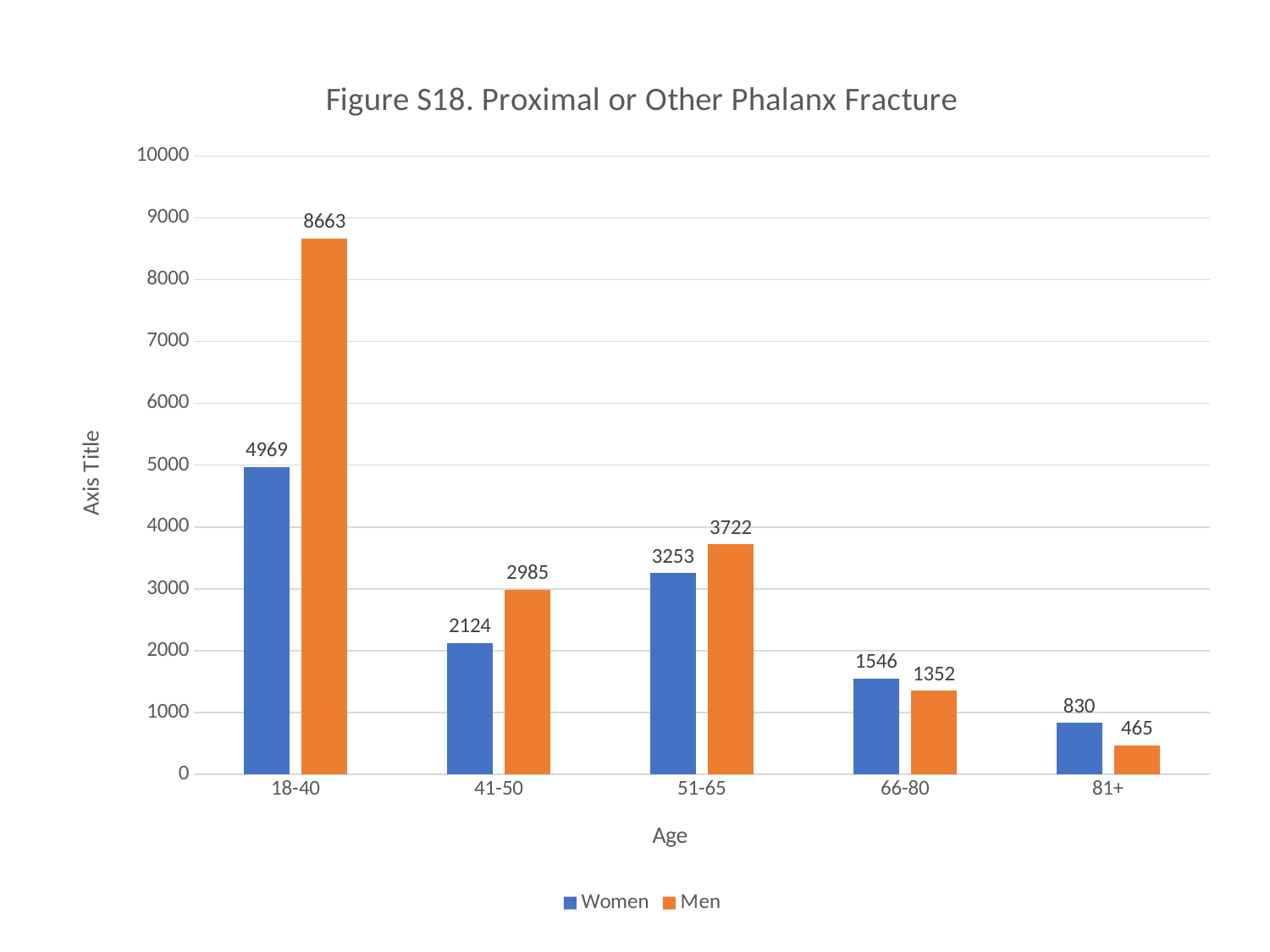

### Chart: Figure S18. Proximal or Other Phalanx Fracture
| Category | Women | Men |
|---|---|---|
| 18-40 | 4969.0 | 8663.0 |
| 41-50 | 2124.0 | 2985.0 |
| 51-65 | 3253.0 | 3722.0 |
| 66-80 | 1546.0 | 1352.0 |
| 81+ | 830.0 | 465.0 |

## Slide 20
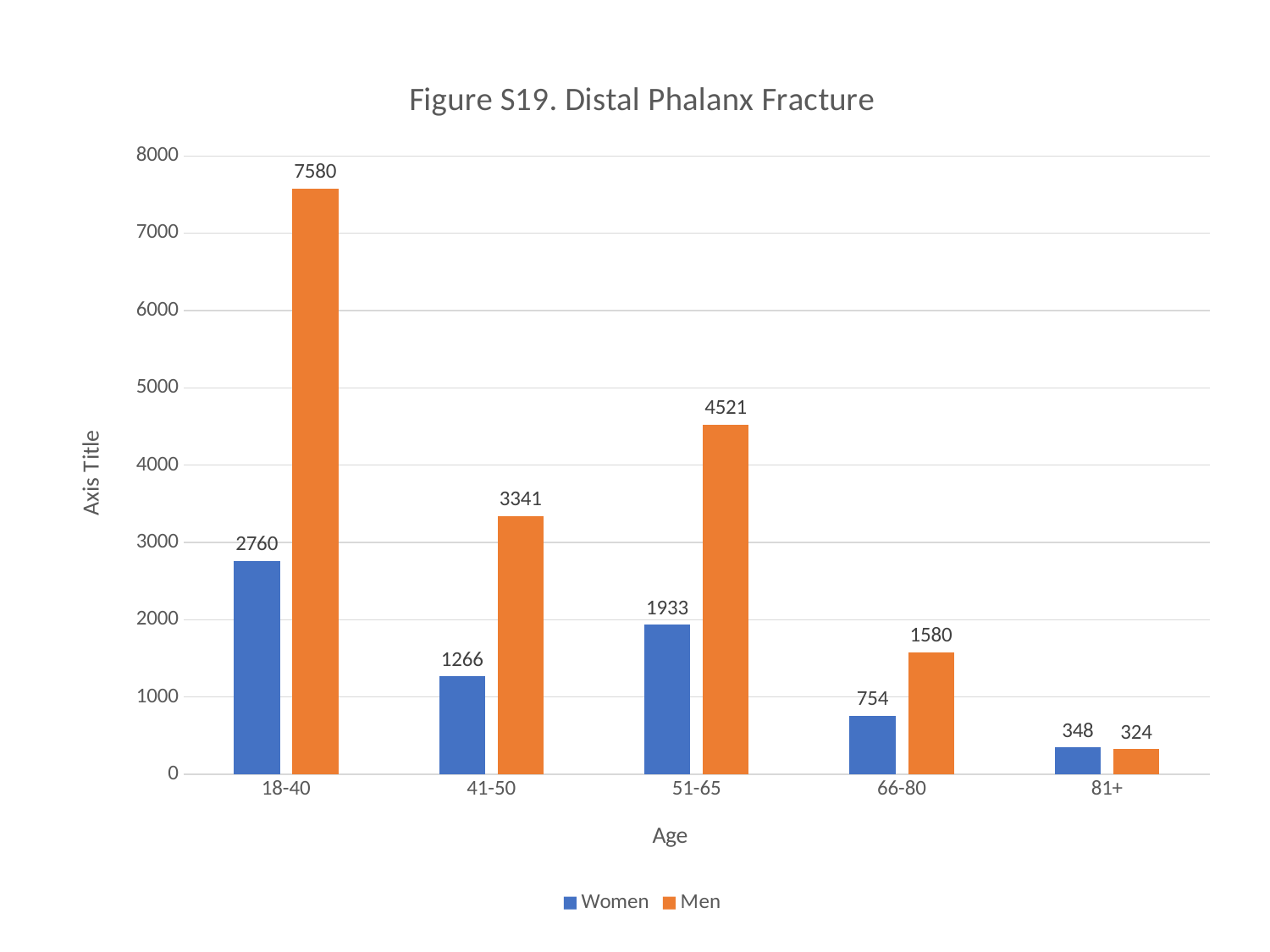

### Chart: Figure S19. Distal Phalanx Fracture
| Category | Women | Men |
|---|---|---|
| 18-40 | 2760.0 | 7580.0 |
| 41-50 | 1266.0 | 3341.0 |
| 51-65 | 1933.0 | 4521.0 |
| 66-80 | 754.0 | 1580.0 |
| 81+ | 348.0 | 324.0 |

## Slide 21
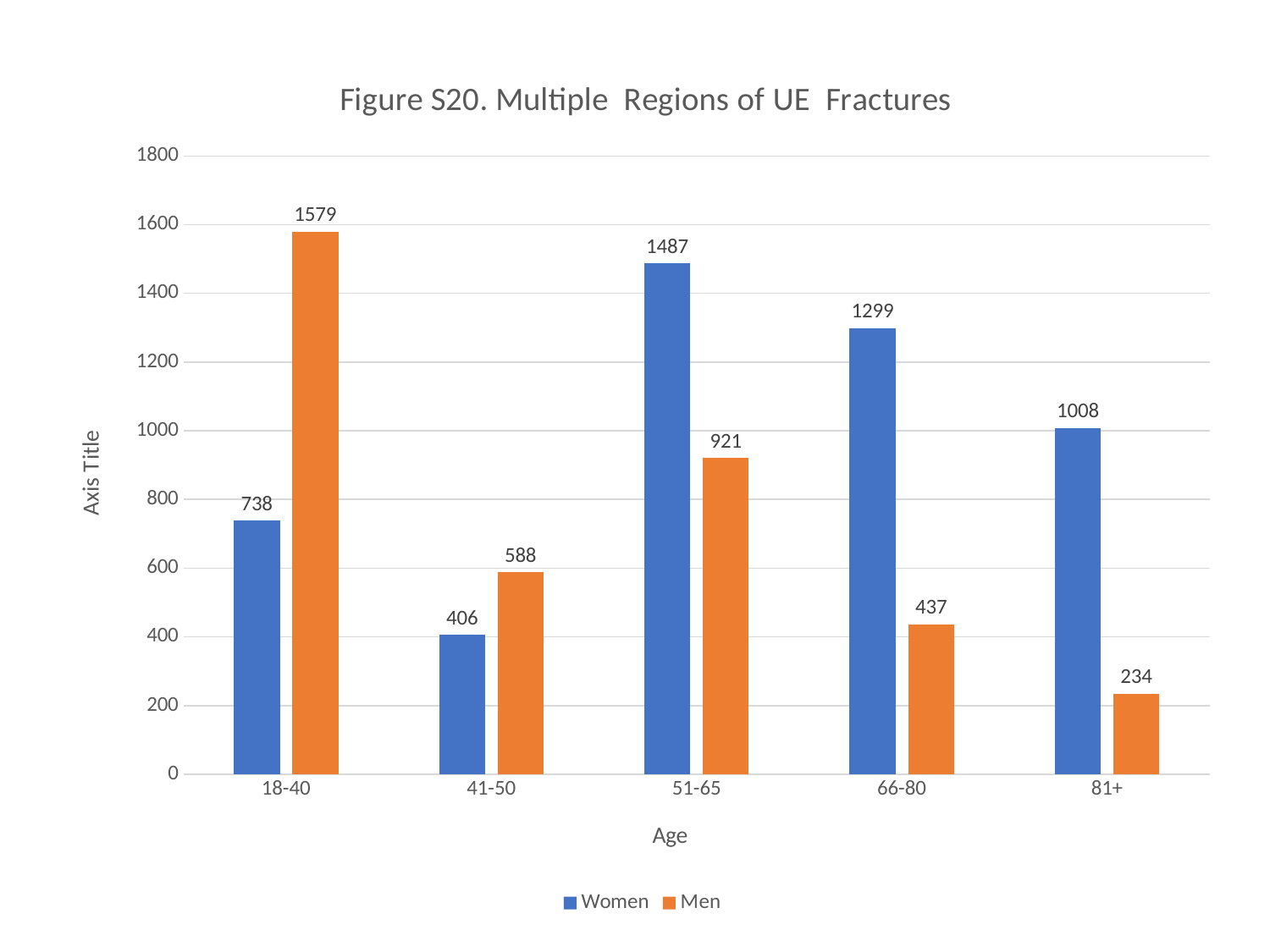

### Chart: Figure S20. Multiple Regions of UE Fractures
| Category | Women | Men |
|---|---|---|
| 18-40 | 738.0 | 1579.0 |
| 41-50 | 406.0 | 588.0 |
| 51-65 | 1487.0 | 921.0 |
| 66-80 | 1299.0 | 437.0 |
| 81+ | 1008.0 | 234.0 |

## Slide 22
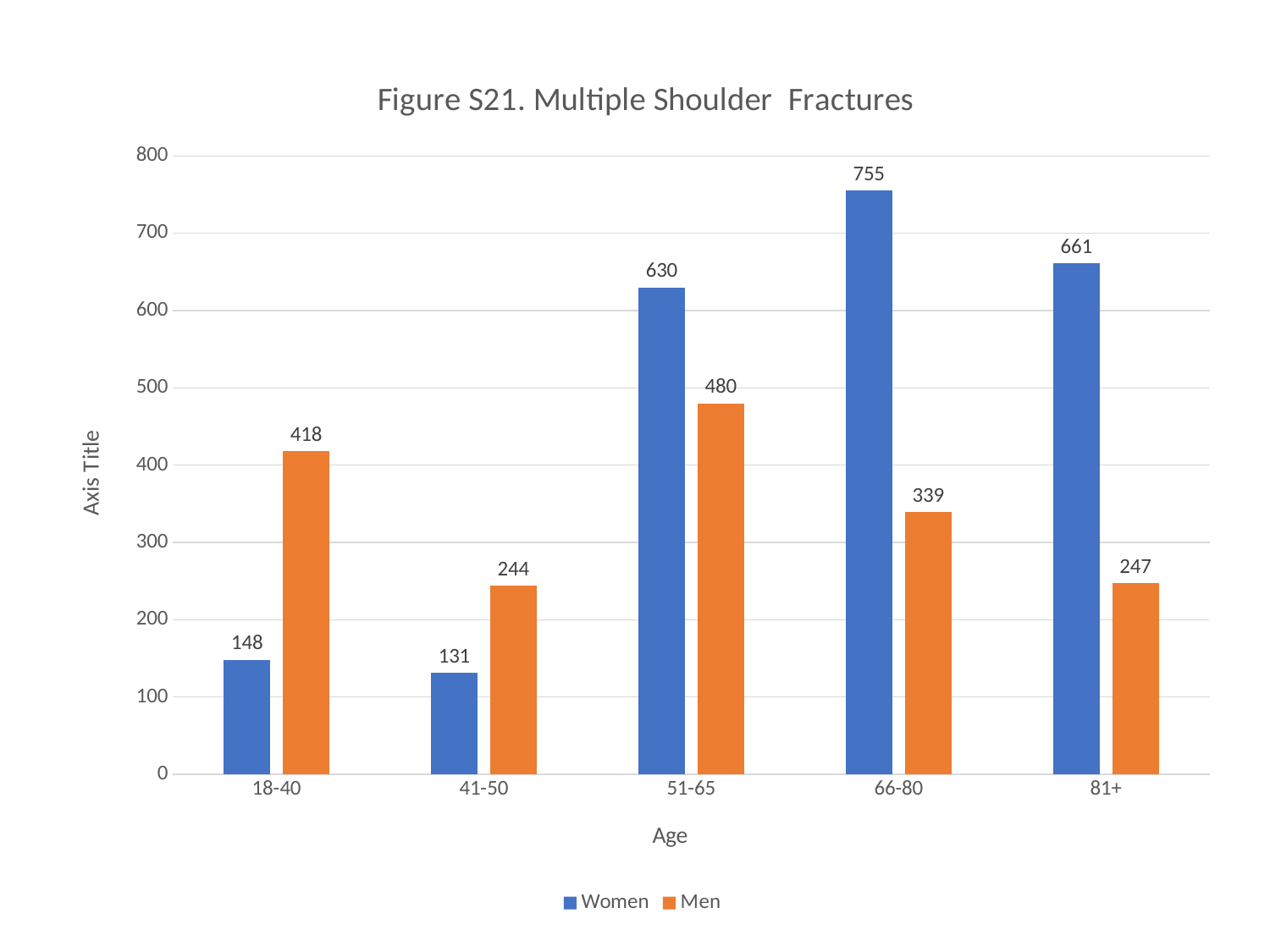

### Chart: Figure S21. Multiple Shoulder Fractures
| Category | Women | Men |
|---|---|---|
| 18-40 | 148.0 | 418.0 |
| 41-50 | 131.0 | 244.0 |
| 51-65 | 630.0 | 480.0 |
| 66-80 | 755.0 | 339.0 |
| 81+ | 661.0 | 247.0 |

## Slide 23
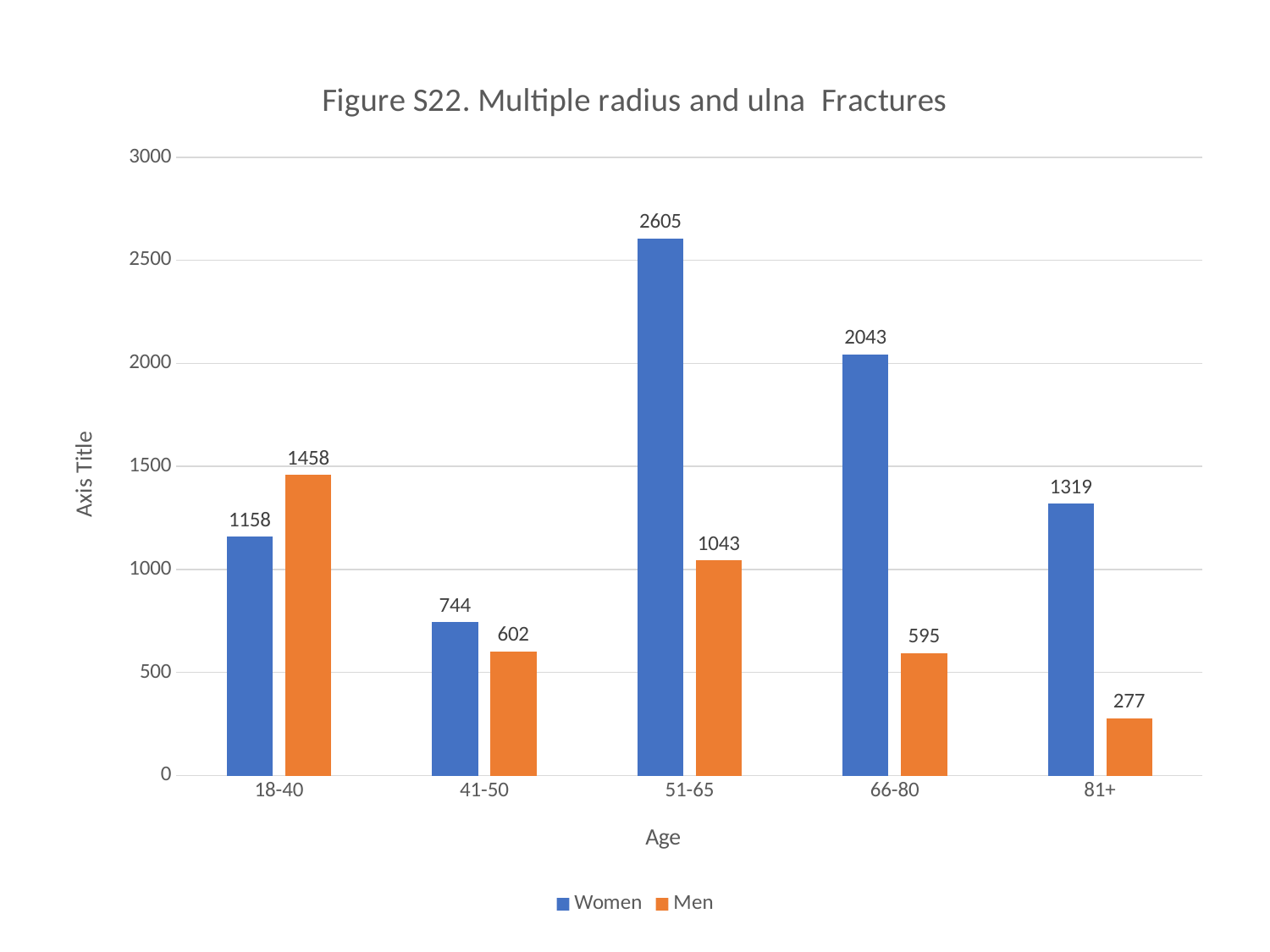

### Chart: Figure S22. Multiple radius and ulna Fractures
| Category | Women | Men |
|---|---|---|
| 18-40 | 1158.0 | 1458.0 |
| 41-50 | 744.0 | 602.0 |
| 51-65 | 2605.0 | 1043.0 |
| 66-80 | 2043.0 | 595.0 |
| 81+ | 1319.0 | 277.0 |
