# Supplementary material for: Fracture profiles of a 4-year cohort of 266,324 first incident upper extremity fractures from population health data in Ontario
Source: BMC Musculoskelet Disord. 2021 Nov 29;22:996. doi: 10.1186/s12891-021-04849-7 (PMC8630866; doi:10.1186/s12891-021-04849-7)
Supplement: Supplementary file 4 — Additional file 4:. (PPTX 145 kb) [file 12891_2021_4849_MOESM4_ESM.pptx]

## Slide 1
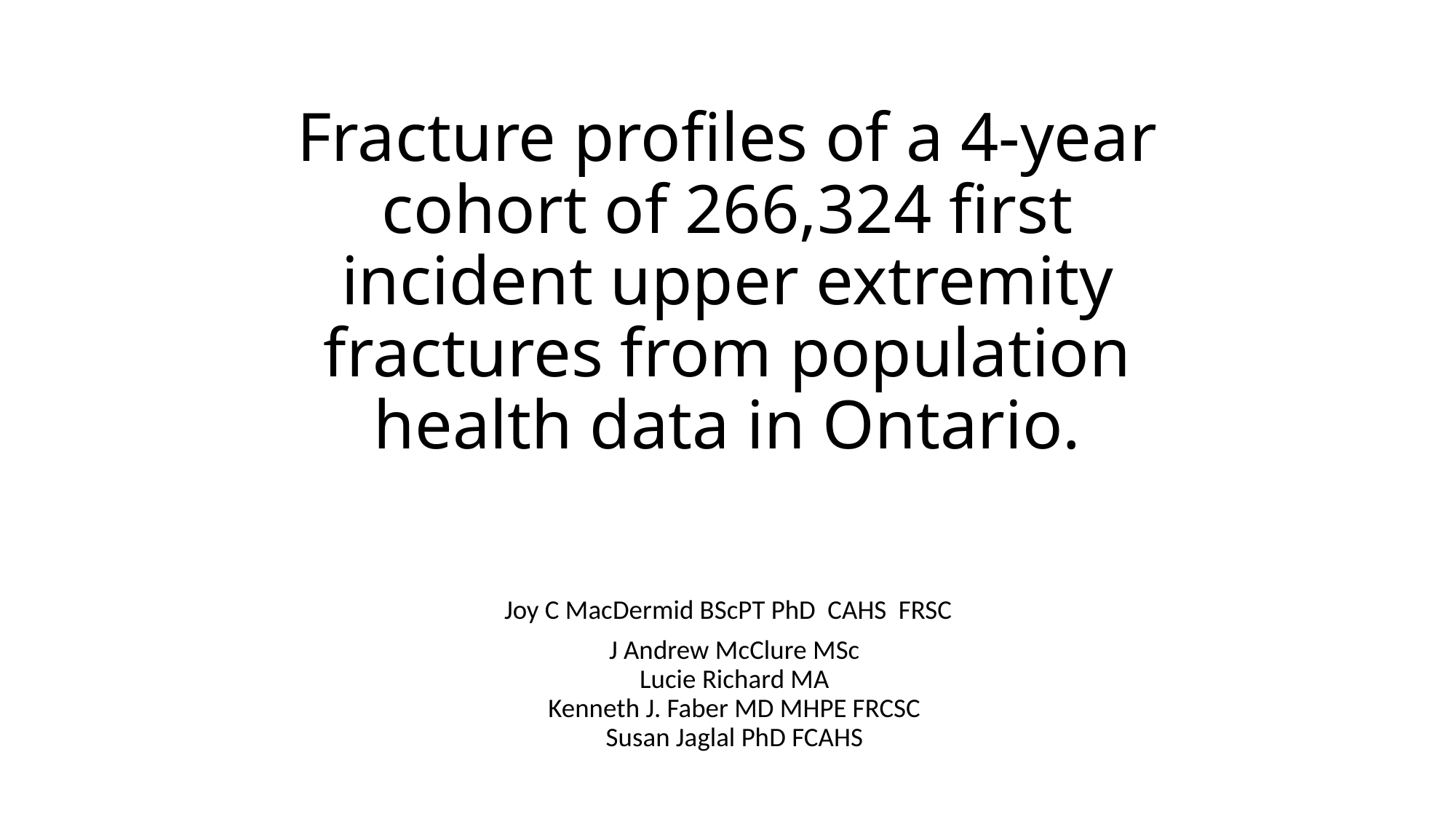

# Fracture profiles of a 4-year cohort of 266,324 first incident upper extremity fractures from population health data in Ontario.
Joy C MacDermid BScPT PhD CAHS FRSC
J Andrew McClure MSc
Lucie Richard MA
Kenneth J. Faber MD MHPE FRCSC
Susan Jaglal PhD FCAHS

## Slide 2
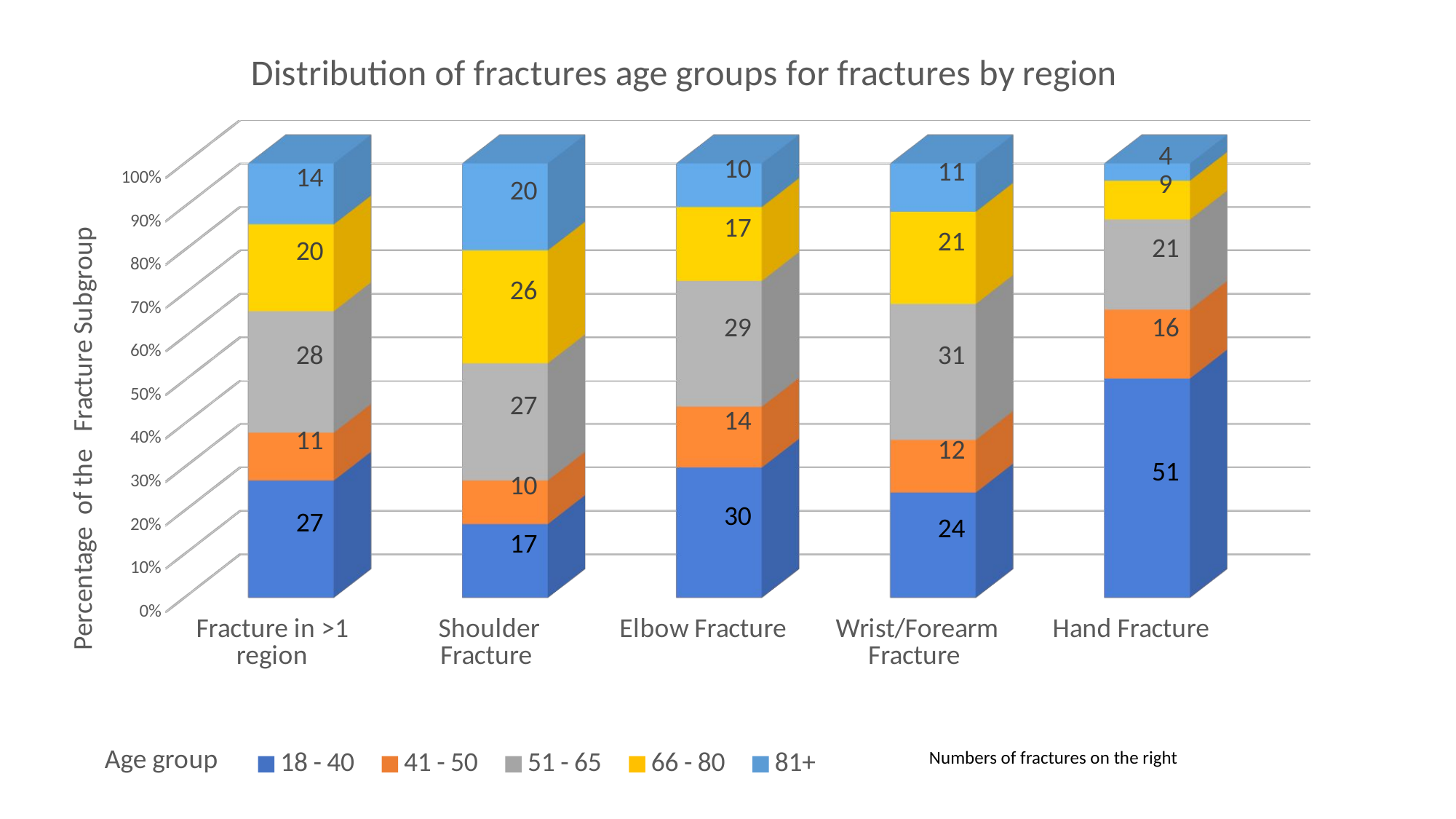

[unsupported chart]
Numbers of fractures on the right

## Slide 3
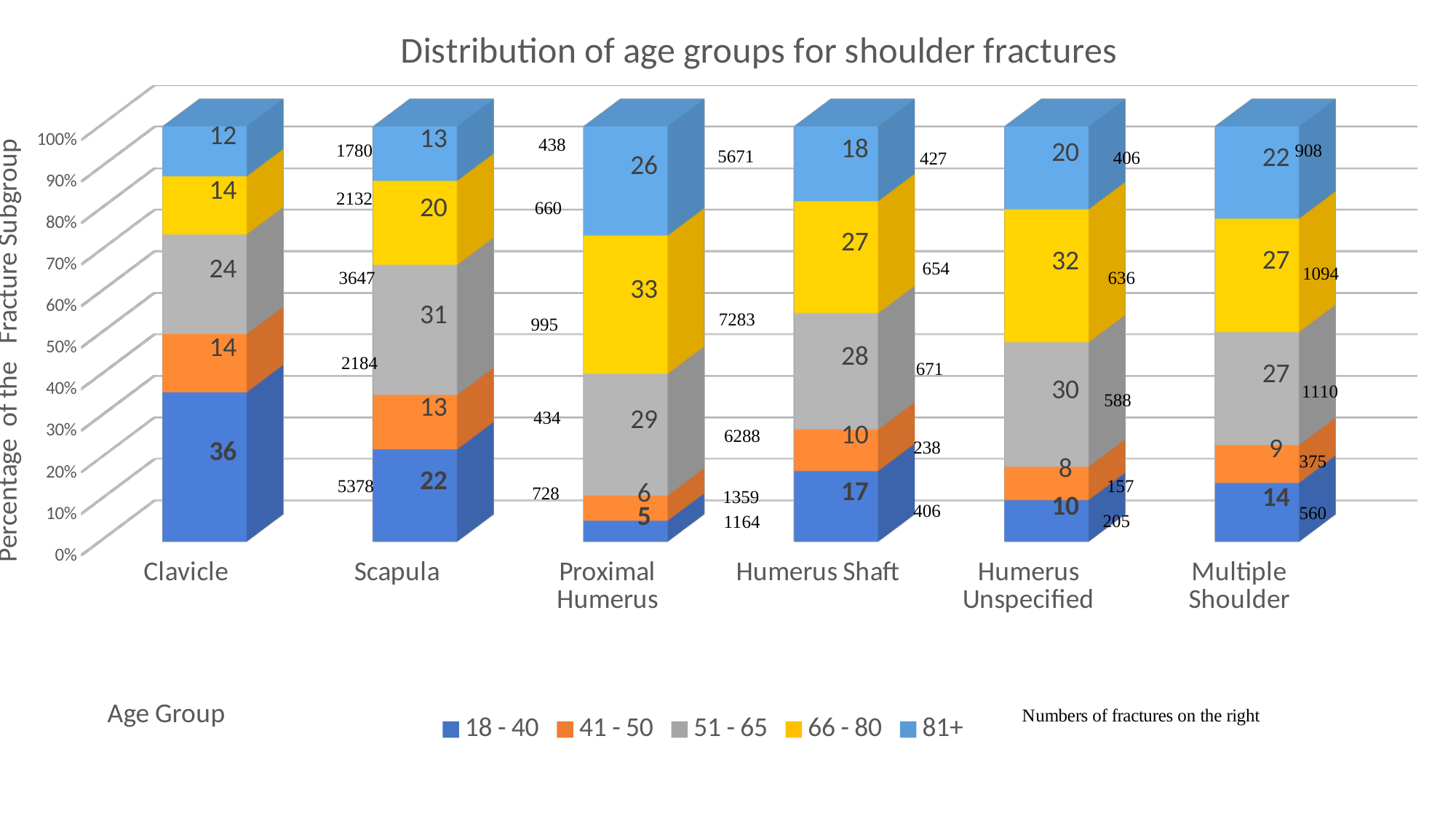

[unsupported chart]

## Slide 4
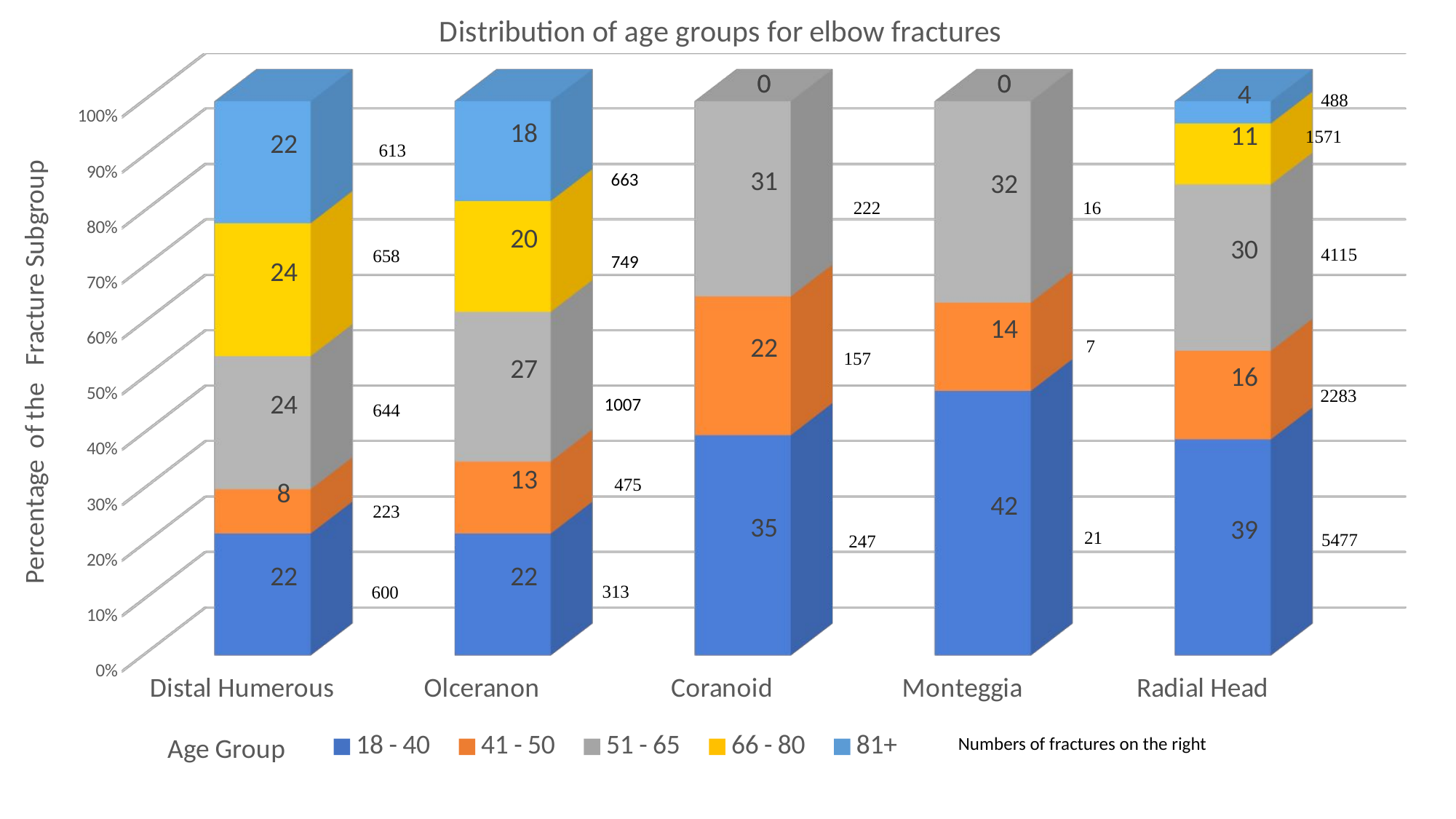

[unsupported chart]
663
749
1007
Numbers of fractures on the right

## Slide 5
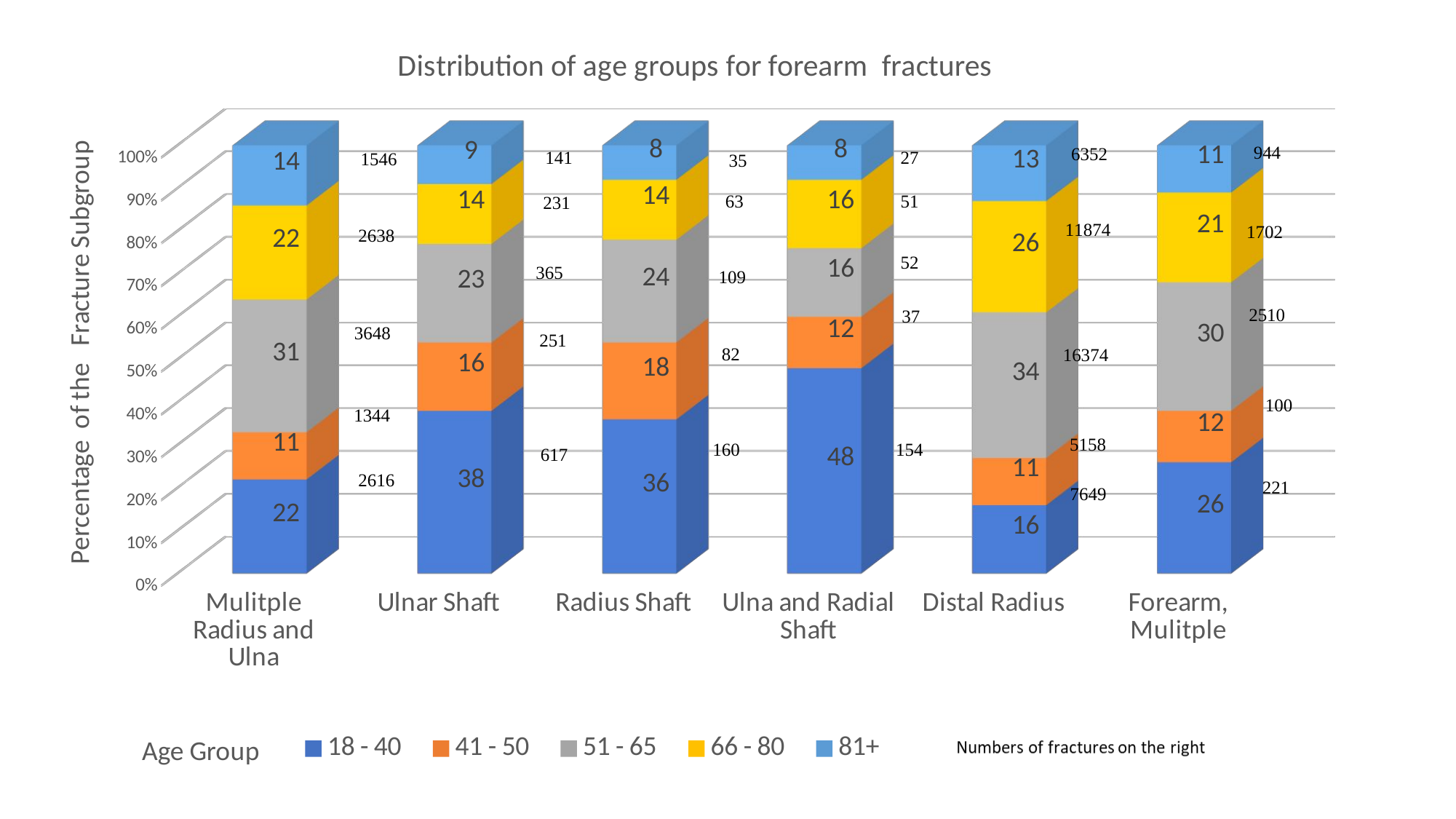

[unsupported chart]

## Slide 6
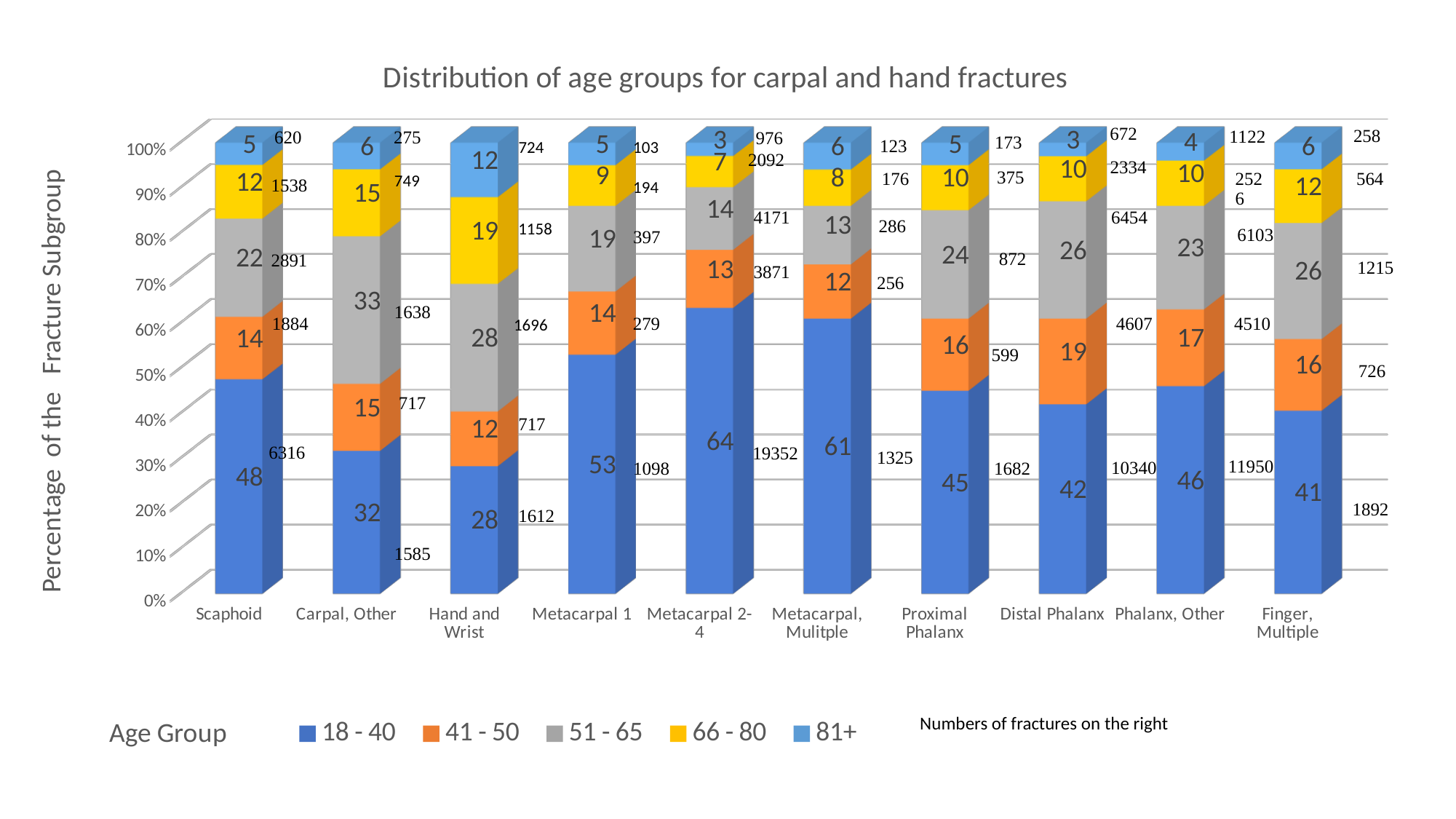

[unsupported chart]
724
103
749
194
1158
1696
Numbers of fractures on the right
